# Nanoscale characterization of PM_2.5_ airborne pollutants reveals high adhesiveness and aggregation capability of soot particles

**DOI:** 10.1038/srep11232

**Published:** 2015-07-16

**Authors:** Yuanyuan Shi, Yanfeng Ji, Hui Sun, Fei Hui, Jianchen Hu, Yaxi Wu, Jianlong Fang, Hao Lin, Jianxiang Wang, Huiling Duan, Mario Lanza

**Affiliations:** 1 Institute of Functional Nano & Soft Materials, Soochow University, 199 Ren-Ai Road, Suzhou Industrial Park, Suzhou, Jiangsu, 215123, China; 2 State Key Laboratory for Turbulence and Complex Systems, Department of Mechanics and Engineering Science, College of Engineering, Peking University, Beijing 100871, China; 3 CAPT, HEDPS and IFSA Collaborative Innovation Center of MoE, Peking University, Beijing 100871, China; 4 Chinese Center For Disease Control and Prevention, Institute of Environmental Health and Related Product Safety. 7 Panjiayuannanli, Chaoyang District, Beijing 100021 China; 5 Department of Mechanical and Aerospace Engineering, Rutgers, The State University of New Jersey, Piscataway, NJ 08854, USA

## Abstract

In 2012 air pollutants were responsible of seven million human death worldwide, and among them particulate matter with an aerodynamic diameter of 2.5 micrometers or less (PM_2.5_) are the most hazardous because they are small enough to invade even the smallest airways and penetrate to the lungs. During the last decade the size, shape, composition, sources and effect of these particles on human health have been studied. However, the noxiousness of these particles not only relies on their chemical toxicity, but particle morphology and mechanical properties affect their thermodynamic behavior, which has notable impact on their biological activity. Therefore, correlating the physical, mechanical and chemical properties of PM_2.5_ airborne pollutants should be the first step to characterize their interaction with other bodies but, unfortunately, such analysis has never been reported before. In this work, we present the first nanomechanical characterization of the most abundant and universal groups of PM_2.5_ airborne pollutants and, by means of atomic force microscope (AFM) combined with other characterization tools, we observe that fluffy soot aggregates are the most sticky and unstable. Our experiments demonstrate that such particles show strong adhesiveness and aggregation, leading to a more diverse composition and compiling all possible toxic chemicals.

Air pollution has become the major problem of many cities, and it is affecting billions of persons^1^,^2^,^3^ around the globe. Among all the noxious pollutants in air, fine particles with an aerodynamic diameter of 2.5 micrometers or less (PM_2.5_) are the most harmful for human health because they are small enough to invade even the smallest airways and penetrate to the lungs^4^. Exposure to this fine particulate matter has been shown to cause respiratory ailments, and can lead to premature death from heart and lung disease^5^. These particles generally come from activities that burn fossil fuels, such as traffic, smelting, and metal processing, which are more abundant in urban areas^6^,^7^,^8^. To give an idea about the magnitude of the problem, while in 2012 the average PM_2.5_ concentration in Los Angeles was 7.4 μg/cm^3^, New Delhi established last year a new record registering 575 μg/cm^3^ (60 times larger than the values considered safe)^9^, and in 2013 Beijing suffered PM_2.5_ concentrations above the limit recommended by the World Health Organization for more than 219 days^10^; according to the Lancet Global Health Burden of Disease Report air pollution is the fourth and sixth cause of death in East and South East Asia^11^ (respectively), and in 2012 seven million people worldwide died due to illnesses linked to air pollution^12^. Moreover, PM_2.5_ pollutants can travel long distances on the atmosphere and may affect surrounding areas. A recent study has reported that Chinese pollution contributed, at a maximum, 12–24% of sulfate concentrations over the western United States^13^.

In the past few years, many studies reported the size, shape, density and source of PM_2.5_^6^,^7^,^8^, as well as their effect in human health^14^,^15^. Despite the morphology and chemical composition of PM_2.5_ may vary depending on the location, some groups of particles are omnipresent and have been invariably reported worldwide. Among them, the most common and abundant are Carbon-rich fluffy soot aggregate from incomplete combustion of hydrocarbons, elongated minerals with high content of metals from coal-fired power plants, and spherical fly ash made of metal-silicates from road dust, construction, coal combustion and secondary atmospheric reactions^6^,^7^,^8^,^16^,^17^,^18^,^19^. Cell viability tests indicate that both organic and inorganic PM_2.5_ components generate oxidative stresses responsible of toxic effects in human health^20^,^21^,^22^,^23^. While inorganic matter has possibly more impact on the respiratory system, organic particles may induce lethal cardiovascular disease^24^,^25^. Although chemical toxicity is the number one health concern, chemistry alone does not determine the noxiousness of the particles, and particle morphology (i.e. shape and surface properties) affect their dynamic behavior and thermodynamic properties, which have notable impact on their biological activity^16^. Therefore, nanomechanical properties determine how the particles morph, stick and aggregate, which strongly affect the way in which they interact with other bodies. However, in-depth nanomechanical particle analyses have never been performed. In this report, we present the first morphological and mechanical analysis of PM_2.5_ and, by means of atomic force microscopy (AFM), we reveal essential information about these particles never reported before, such as surface roughness, stickiness, deformation and elasticity. Further scanning electron microscope images (SEM) and energy dispersive X-ray spectroscopy surveys (EDAX) allowed to link these properties to the chemical composition and, therefore, to the sources. We analyze the most abundant and universal groups of PM_2.5_ airborne pollutants and, after thoroughly investigating more than 500 fine particles with different tools, our results indicate that PM_2.5_ soot aggregates exhibited the largest surface roughness, stickiness and deformation. Such observation is indeed indicating that these particles, not only can easily compile all other toxic chemicals, but also are difficult to remove when interacting with other bodies, which may cause longer exposure. This general behavior has been statistically corroborated processing experimental data through Weibull analytical models.

## Results And Discussion

**Figure 1:**
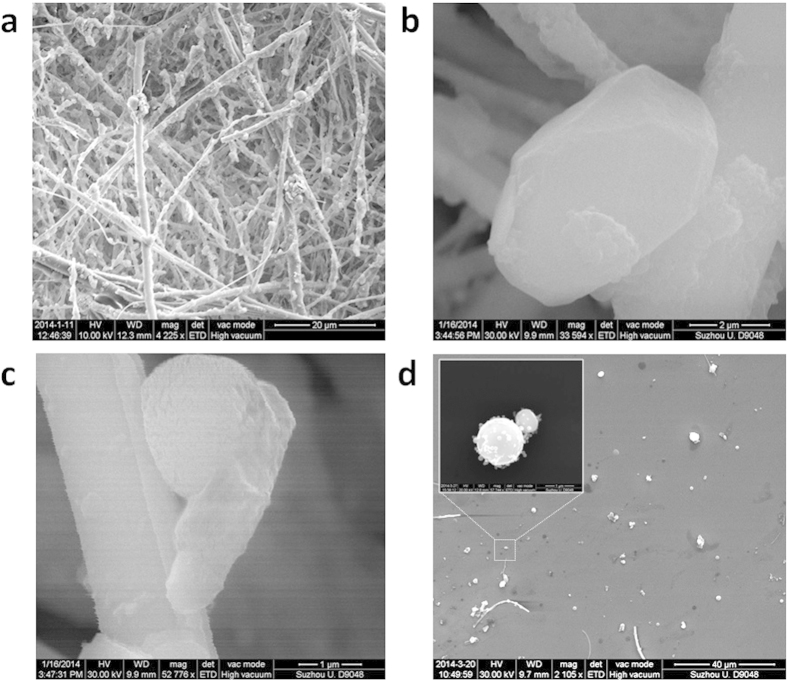
Morphology of PM_2.5_ particles. (**a**) large area image of as-collected PM_2.5_ on the filamentary filter. (**b**) and (**c**) SEM images of a particle with flat and rough top surface, respectively. (**d**) SEM image of the PM_2.5_ transferred on a Silicon substrate. Inset, zoom-in SEM image of an Iron-rich particle. The scale bars are 20 μm for image (**a**) 2 μm for image (**b**) 1 μm for image (**c**) 40 μm for image (**d**).

PM_2.5_ particles have been collected on December 2^nd^ and 7^th^ of 2013 in Beijing, following the regulations established by the US Environmental Protection Agency. For the particle collection, we used the TH-150C Automatic Medium Volume TSP Sampler coupled with TH-PM_2.5_, which is strategically designed to retain in the filters only small particles with diameters below 5 μm (see supplementary information). The initially white filters become darker depending on the degree of pollution of each day, and the particles can be easily detected with the optical microscope (Figures S1 and S2 in the supplementary information). Figure 1a shows the typical SEM picture of a contaminated filter: a dense network of filaments with incrusted pollutant particles, most of them PM_2.5_. From all detected particles, we focus only on those with an aerodynamic diameter smaller than 2.5 μm and, at a first stage, we analyze their size and composition as collected in the filters - one by one - from SEM pictures and EDAX surveys, respectively. The PM_2.5_ exhibited circular, elongated and irregular shapes, as well as high densities of O, Si, C, Fe, Ca, Mg, Al, K, and S (Figures S3, S4 and S5). We start our novel analysis by studying the surface roughness of PM_2.5_. It is widely known that surface roughness can remarkably influence the physical and mechanical properties of a body^26^. Figure 1b,c show the typical example of PM_2.5_ particles with flat and rough surfaces (respectively, see also Figure S6). It is worth noting that, while the particle with a flat surface has a well-defined shape, the rough one seems to adapt to the shape of the filament network in the filter, suggesting that it could be easily deformed. When a body can deform its shape to adapt to the contour of another, the contact area between them increases, which enhances their adhesion force (capillary forces, Wan der Waals, electronic attraction)^27^.

We analyze the capability of PM_2.5_ to adhere to other bodies and, to do so, the surface properties of the particles are in-depth analyzed by means of AFM and related modes. AFM maps are very powerful to analyze the properties of the particles because they can provide three dimensional information with very high lateral and vertical resolutions. But the main problem is that the filamentary network of the filters used to collect PM_2.5_ doesn't allow reliable AFM analysis, it is too rough^19^,^28^. Other authors used polycarbonate^6^, quartz^7^ and mica substrates^29^ but, even if those materials don't present a dense network of filaments, their roughness is still too large, which may lead to confusing images with the AFM. To solve this problem we developed a method to transfer the particles to arbitrary substrates by sonicating the filters together with a target substrate in the absence of solvent (Figure S8), using a low power (40 W), frequency (40 KHz ± 10%) and short times (1–40 min). The target substrate consists of cleaned Silicon wafers (Figure S7), and allowed direct correlation between AFM and EDAX data, and vice versa. Figure 1d shows an SEM picture of PM_2.5_ particles transferred on the Silicon substrate (also in Figure S9); the inset shows the zoom-in image of an Iron- and Oxygen-rich particle similar to those classified as fly ash^6^,^8^. We corroborate that the particles under test are representative of the well-established universal groups of PM_2.5_. Figures S10, S11 and S12 in the supplementary information show the shape and chemical composition of the particles in this study and in the literature for the main three groups analyzed: fluffy soot aggregate, elongated minerals with high content of metals and spherical fly ash made of metal-silicates. The striking similarity between the particles in this work and those previously reported worldwide establishes the immediate importance and relevance of our study. We further analyze the reliability of this method comparing the types of particles observed in three different samples: i) in-situ collected on the filter (Fig. 1a–c and S3–S5), ii) collected on the filter but transferred to the Silicon (Fig. 1d and S10–12), and iii) in-situ collected on a piece of Silicon directly introduced in the collector (Figure S13). We observed a larger density of small particles in in-situ experiments (diameter below 500 nm, see Figure S14a), while after the transfer the particles measured were slightly larger (diameter usually above 500 nm, see Figures S10–S12). Such observation has two implications. The first is that the transfer process didn’t fragment the particles. The absence of solvent minimizes particle fragmentation, and only the vibrations necessary to detach the particles from the filter are generated, so that they can precipitate on the target substrate^30^. And the second is that smallest particles couldn't be transferred. We further analyze those particles with SEM and EDAX, and observed that their surface and chemical composition is very similar to those larger transferred on the filter (Figure S14), indicating that they are not a different type of particle, but just smaller pieces of soot, elongated minerals and fly ash. It is worth noting that some atmospheric particles can be semi-to-entirely liquid when they are airborne, which would difficult the filter-to-Silicon transfer process. Interestingly, we also observe many particles surrounded by a dark layer on both in-situ (Figure S14d) and after transfer (Figure S28). Due to its regular contour and flat shape, this dark trace may be related to the semi-liquid nature of some particles. Such observations corroborate that we successfully transferred most classes of PM_2.5_ airborne pollutants collected on the filters (without altering them).

**Figure 2:**
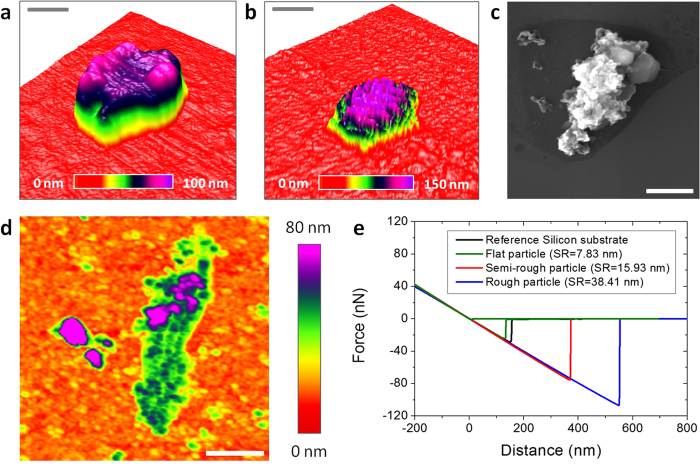
Mechanical properties of PM_2.5_ particles measured with AFM. (**a**) and (**b**) three dimensional topographic AFM images of similar PM_2.5_ particles with flat and rough surfaces respectively (on Silicon substrate); the AFM is able to display the surface roughness with a precision of Angstroms. (**c**) and (**d**) SEM and topographic AFM images of PM_2.5_ soot aggregate with high surface roughness (respectively). (**e**) Example of force-distance curves measured on particles with different surface roughness (SR); the negative peak of the backward curve corresponds to the AFM tip-sample adhesion force; particles with higher surface roughness usually exhibited larger adhesion forces. The scale bars are 300 nm for (**a**) and 2.5 μm for (**b**) 3 μm for (**c**) 1.1 μm for (**d**).

Once the particles rest on flat Silicon, performing accurate AFM research is feasible, and therefore, we scanned the surface of the PM_2.5_-rich Silicon substrate in tapping mode (Figure S15). The AFM characterization has been intentionally performed in air environment to monitor the real properties of the particles in atmosphere. Kollensperger *et al.*^29^ demonstrated that the exposure of PM_2.5_ pollutants to different relative humidities could alter their surface composition. Depending on their wettability, airborne pollutants could retain a water layer at the surface, which could alter the intrinsic adhesiveness of the particles measured by AFM due to the addition of capillary effects^27^,^31^. Therefore, using vacuum AFM may lead to false imaging of such properties, due to the removal of the natural water layer on the particles^32^,^33^. During our AFM experiments the temperature and relative humidity of the room ranged between 6–10 °C and 60−70% (respectively). Figure 2a,b show the three dimensional AFM topographic images of PM_2.5_ particles with flat and rough surfaces (respectively). The extraordinary high vertical resolution of the AFM allows displaying the rugosity of the PM_2.5_ surface with an unprecedent precision of Angstroms (correlated SEM images in Figure S16). Using the NanoScope Analysis software of the AFM we have been able to easily and accurately calculate the surface roughness of each particle (Figures S17 and S18), and values of 14.60 nm and 39.51 nm for the particles in Fig. 2a,b have been obtained (respectively). Moreover, off-line process of the images using the AFM software facilitate the determination of the diameter and area of the PM_2.5_, something that is much more laborious to do from SEM images. The resulting statistical analysis of the particle diameter shows a maximum at 1.34 μm (Figure S19). It is worth noting that, the physical diameter measured in SEM and AFM images may not necessarily correspond to the aerodynamic one and, therefore, some particles with a physical diameter below 2.5 μm may could not strictly be considered PM_2.5_. The relationship between the aerodynamic diameter, physical diameter and particle density can be expressed as:


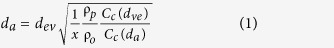


where, χ is the dynamic shape factor (in any flow regime), d_a_ is the aerodynamic diameter, d_ve_ is the volume equivalent diameter, C_c_ is the Cunningham slip correction factor, ρ_p_ is the particle density and ρ_o_ is the standard density (1 g/cm^3^)^34^. Different methodologies to measure the particle density have been presented in the past^35^,^36^,^37^,^38^,^39^,^40^,^41^,^42^. For nonspherical particles, most techniques provide "apparent" or "effective" densities which, as stated in DeCarlo *et al.*^34^, could be not necessarily a true measure of the particle. In any case, the complexity of these techniques makes the calculation of the particles density to be out of the scope of this study. Despite filtering the particles only by diameter may induce some degree of error, we believe it is small and we could evaluate it. Previous PM_2.5_ studies reported particle densities mainly between 1 g/cm^3^ and 3 g/cm^3^ 40,41. As the particle density in Eq.1 is inside the square root, a maximum increase of density (ρ_p_) u_p_ to 3 g/cm^3^ would produce an increase of 1.73 in the aerodynamic diameter (d_a_). That would imply that, for a maximum particle with a density of 3 g/cm^3^, a physical diameter below 1.44 μm would ensure an aerodynamic diameter of 2.5 μm or less. Interestingly, in the statistical analysis of the diameter (shown in Figure S19) most of the particles we analyzed are below 1.44 μm in diameter (the maximum peak of the distribution is at 1.34 μm). Despite a remarkable amount of particles with physical diameters between 1.44 and 2.5 μm are analyzed in our work, it is also reasonable to think that not all particles will reach the maximum densities above mentioned. Furthermore, Kelly and McMurry^42^ and Schleicher *et al.*^38^ observed that the calculated densities of aggregates were much lower than the bulk density of the particle material. Therefore, since many of the largest particles analyzed in this study may be aggregates (with diameters between 1.4 and 2.5 μm, see Figures S10−S12), in this work we will rely on the physical diameters measured in the SEM and AFM images, as many other authors did in the past^6^,^8^,^19^.

**Figure 3:**
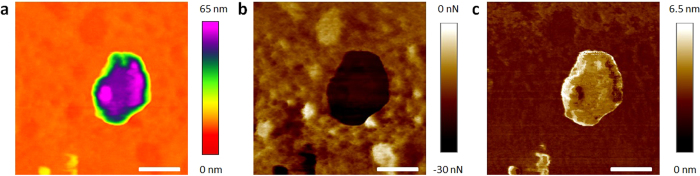
Adhesion and deformation of PM_2.5_ particles. (**a**) (**b**) and (**c**) topographic, contact force and deformation maps of a PM_2.5_ particle with a flat surface (respectively). The images reveal a more complete picture of the mechanical properties of the particle. Since many particles can be analyzed during the same scan, this AFM mode paves the way to statistically analyze the mechanical properties of the particles. The scale bars are 300 nm for (**a**–**c**).

Subsequently, the mechanical properties of airborne pollutants with a physical diameter below 2.5 μm (most of them PM_2.5_) were analyzed by means of AFM topographic maps and Force-Distance (F–Z) curves in contact mode. The shape of the force-distance (F–Z) curves in contact mode can provide information about tip/particle adhesion, particle deformation, elasticity, rupture and indentation^43^,^44^. It should be noted that such experiment has never been performed before, and all previous AFM studies mostly analyzed the particles by topographic/phase maps in tapping mode. The main reason is that, in contact mode, conventional AFM tips may move the particles on the surface of the substrate, leading to false imaging. To avoid this problem, we use specific AFM tips with a very long cantilever (450 μm) and low spring constant (0.2 N/m) from Bruker (see methods). Using this methodology, we are able to monitor the intrinsic topography of the samples without introducing external modification. As an example, Fig. 2c,d show the SEM and AFM images (respectively) of a PM_2.5_ soot aggregate (see EDAX composition analysis in Figure S20). As Fig. 2d reveals, all the tiny features of its characteristic fluffy surface can be clearly observed with AFM. By sequences of topographic maps measured at the same location using different contact forces, we analyze the deformation and elasticity of the particles. As an example, the particle in Fig. 2d exhibited shape changes, increasing its length and decreasing its height, as displayed in successive sections (Figure S20). Further AFM maps didn't reveal any other shape change, indicating that the particle cannot recover its initial shape, leading to plastic deformation (elastic deformation and shape recovery has been rarely observed). We also analyze the stickiness/adhesiveness of the particles by F−Z curves. Figure 2e shows the F−Z curves collected on particles with different surface roughness (the F−Z curves measured on bare Silicon are also shown as reference). The adhesion forces measured on flat particles are ~20 nN respectively, close to the values of non-sticky flat surfaces such as Silicon, glass and aluminum; on the contrary, rough particles showed adhesion forces above ~100 nN, a stickiness comparable to that of scotch tape or laboratory Carbon tape (Figure S21). A low percentage of ultra-sticky PM_2.5_ rich in Carbon may attach to the AFM tips (Figure S22). The adhesion and deformation of the particles have been also analyzed by scanning the surface of the samples with the Peak Force Quantitative Nanomechanical Mapping (QNM) tool from Bruker Dimension Icon AFM (see methods), which provides information at each pixel of the image (Figure S23). As an example, Fig. 3a–c show the topographic, adhesion and deformation maps (respectively) of a typical PM_2.5_ particle with a flat surface. The negative contact force in Fig. 3b indicates adhesive force, i.e. the particle shows an adhesion force of 25 nN (contact force of −25 nN). As it can be observed, the values of adhesion force and deformation at each image deviate from the maximum/minimum values detected on the flat Silicon, and local differences can also be detected. More specifically, the particle displayed in Fig. 3 holds a very flat surface with more progressive height changes than local fluctuations, which interestingly, produces an almost constant adhesion force. Such behavior has been observed in particles with regular shapes and fine surfaces, while rough morphologies normally showed larger deviations. The genuine capability of this tool to analyze the adhesiveness and deformation of many particles during the same scan allows for the first time the statistical analysis of these properties.

**Figure 4:**
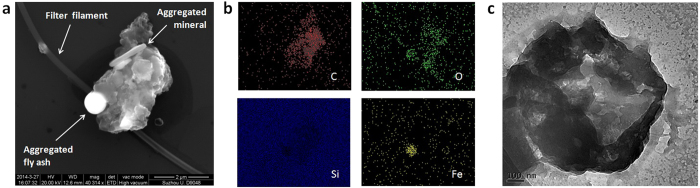
Aggregate of PM_2.5_ particles. (**a**) and (**b**) SEM and EDAX analyses of a PM_2.5_ particles aggregate on clean Silicon; the rough Carbon-rich soot particle shows to be very sticky and can attach Iron-rich spherical fly ash, elongated minerals and even rip off filter filaments during the transfer. (**c**) TEM analysis of an organic particle showing hierarchical structure and proving the aggregation of small flakes and nanoparticles. The scale bars are 2 μm for (**a**) and 100 nm for (**c**).

**Figure 5:**
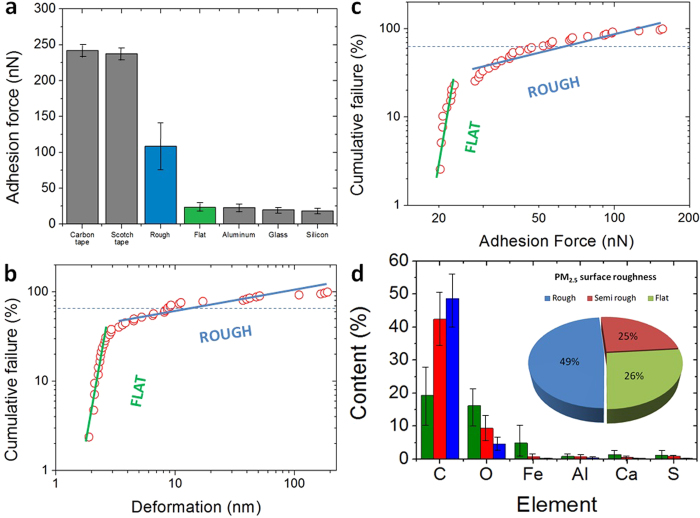
Relationship between mechanical properties and chemical composition of PM_2.5_. (**a**) Adhesion force of PM_2.5_ with rough and flat surfaces compared to standard sticky and non-sticky substrates. (**b**) Weibull probability plot of the adhesion force collected on two groups of PM_2.5_ particles (with low and high surface roughness); (**c**) statistical analysis of the deformation of the same particles; both studies corroborate the different mechanical properties of rough and flat PM_2.5_ particles, and stickier particles usually show a larger deformation. (**d**) EDAX Chemical composition histogram of the particles collected with SEM/EDAX classified by surface roughness; a larger surface roughness (and therefore, stickiness and deformation) link to a larger content of Carbon, while particles with a flat surface (low stickiness and viscosity) are richer in Oxygen and metals.

The large stickiness of some PM_2.5_ has been also proved from SEM/EDAX analysis. Fig. 4a,b show the SEM and EDAX images of a sticky PM_2.5_ soot particle. As it can be observed, the rough particle made of Carbon can aggregate other with typical shapes and chemical compositions, such as Iron- and Oxygen-rich fly ash and elongated minerals. The ability of fluffy soot particles to aggregate the rest has been repeatedly observed (Figure S24), which could increase the toxicity of the particle. Some particles are so sticky that they can even rip out some filter filaments during the transfer. The aggregation capability of soot PM_2.5_ has also been observed from Transmission Electron Microscope (TEM) images (Fig. 4c), which indicate that the sticky nature of these pollutants leads to a hierarchical structure made of thin layers and small particles aggregated on them. Compared to the reference substrates, fluffy-rough particles showed larger adhesion forces in F–Z curves (Fig. 5a), which is responsible of nanoparticle aggregation. The PM_2.5_ aggregates analyzed in this study systematically showed large densities of Carbon (Figures S23, S24 and S25). Smith *et al.*^17^ studied the PM_2.5_ in London air, and suggested that organic pollutants may tend to aggregate, while pure metals and minerals do not. The larger adhesiveness and deformation of fluffy soot aggregate particles have been statistically corroborated through the Weibull probability plots (Fig. 5b,c). The mass mobility and fractal dimension of soot aggregate is especially important because it remarkably affects the way in which it interacts with other bodies, and hence, the exposure experienced by the receptor^16^. The lower slope of the Weibul plots for fluffy-rough particles rich in Carbon (soot aggregates) reveal a larger variability of these characteristics, indicating that they are more unstable from a mechanical point of view. On the contrary, particles with a flat surface and rich on stable metallic oxides and silicates (fly ash and elongated minerals) exhibited lower and uniform stickiness and deformation. The larger content of Carbon of rough, sticky and deformable particles (soot aggregates) has been statistically corroborated with EDAX (Fig. 5d). We quantified the amount of particles of each type and concluded that, in the more than 500 particles analyzed: 49% are rich on Carbon, sticky, deformable and unstable, 26% showed larger stability and were composed of metallic oxides, and the remaining 25% showed an intermediate behavior. The first group can be clearly linked to fluffy soot aggregate, while the last two groups correspond to elongated minerals and fly ash indistinctly. These values are comparable to those previously reported^16^,^17^,^18^,^19^,^45^. Finally, we observe that 54% of the PM_2.5_ particles analyzed appeared surrounded by a dark area in the SEM images. We analyze the morphology of such layer by means of AFM and observed that such areas are flat plateaus with thicknesses between 2 and 10 nm (Figure S28). Despite EDAX analyses were unable to assess the chemical composition of these plateau-like areas, the observation of regular contours make us think that such layer may be formed by semi-liquid particles. The observation of much more particles surrounded by this layer in in-situ experiments on Silicon further supports this hypothesis: semi-to-entirely liquid particles may be difficult to transfer from the filters to the flat Silicon substrate. We further analyzed the stickiness of this plateau-like layer by means of, AFM F–Z curves and adhesion/deformation maps, but did not reveal any increase of the adhesion force, suggesting the formation of an inert layer. Probably Oxygen atoms from the PM_2.5_ particle may interact with the underlying Silicon substrate leading to the formation of a thin SiO_2_ layer^46^. Such oxidation may be responsible for some of the toxic effects of PM_2.5_ on human cellular tissue^8^. Future cell viability assays should conduct to clarify of the degree of toxicity of these specific particles.

## Conclusion

In conclusion, we statistically analyzed the physical and chemical properties of the most representative and universal groups of PM_2.5_ airborne pollutants by means of AFM, SEM, TEM and EDAX. After a thorough characterization of more than 500 particles, our study reveals that fluffy soot particles show the capability to retain other species with different sizes and chemical compositions, increasing the chemical toxicity of the resulting aggregate. Our unprecedented AFM analysis revealed that such capability is related to the intrinsic high adhesiveness of soot aggregates. The experimental methodologies used here, including a method to transfer PM_2.5_ from rough collection filters to flat analysis-friendly substrates, pave the way for future in-deep nanoscale characterization of airborne pollutants.

## Methods

In this investigation, we developed a method to transfer PM_2.5_ particles on flat substrates for friendly physical and mechanical characterization with Atomic Force Microscope. A piece of polluted filter and cleaned Si substrate were placed together in a plastic tube box; the tube was then immersed in a glass with water and sonicated for different times to let the particles precipitate on the Silicon (see supplementary information). The relationship between surface roughness and chemical composition was studied by using the Quanta 200FEG Scanning Electron Microscope coupled with Energy Dispersive X-ray Spectrometer (SEM-EDAX). High-resolution Transmission Electron Microscope (HRTEM) images were obtained with the Tecnai G2 F20 using standard copper grid and a field voltage of 20 KV. The mechanical properties of the particles were analyzed using two kinds of AFM: the Multimode V AFM and Dimension Icon AFM form Bruker. The first one was used to measure F–Z curves in contact mode using standard commercially available AFM tips from Bruker (model SCM-PIC). These tips were made by Silicon micromachining and were coated first with a 20 nm thick layer of Pt–Ir, which is a 95% platinum and 5% iridium alloy (the iridium is used to enhance the stability of the platinum layer). The other main characteristics of these tips are: thickness = 2 μm, width = 50 μm, length = 450 μm, spring constant = 0.2 N/m, resonance frequency = 13 kHz and nominal tip radius = 20 nm. The Dimension Icon AFM was used to measure adhesion and deformation maps. The images are built from the information collected at each point (which represents the value of each pixel of the map (see supplementary information). For these experiments Co-Cr coated Silicon tips from Burker were used. The main characteristics of the tip are: thickness = 1.85 μm, width = 30 μm, length = 125 μm, spring constant = 5 N/m, resonance frequency = 150 kHz and nominal tip radius = 35 nm. The maps were recorded using a scan frequency of 1 Hz, and a drive amplitude of 5 V. All AFM data were analyzed with the NanoScope Analysis AFM software from Bruker (version 1.40), and the plots were generated with OriginLab 8.0.

### Associated content

Supporting Information available: PM_2.5_ collection process, Digital camera pictures of the contaminated filters, Optical microscope pictures of the contaminated filters, SEM-EDAX study of most representative particles on the filters, Methodology to select particles with rough and flat surfaces from SEM images, Silicon substrates used in this investigation, Process to transfer PM_2.5_ from rough filters to flat analysis-friendly substrates, Representative SEM images of PM_2.5_ transferred on Silicon, Representative AFM images of PM_2.5_, transferred on Silicon, Correlation between AFM and SEM images, Quantification of the particles roughness with AFM, Statistical analysis of PM_2.5_ size transferred on Silicon, Universal groups of PM_2.5_ airborne pollutants, Analysis of PM_2.5_ deformation from topographic AFM maps, Values of adhesion force in reference substrates, Particle adhesion to the AFM tip, Measurement of the adhesion and deformation with AFM, Ability of Carbon-rich soot to aggregate other particles, Effect of some of PM_2.5_ to surrounding areas.

## Additional Information

**How to cite this article**: Shi, Y. *et al.* Nanoscale characterization of PM_2.5_ airborne pollutants reveals high adhesiveness and aggregation capability of soot particles. *Sci. Rep.* **5**, 11232; doi: 10.1038/srep11232 (2015).

## Supplementary Material

Supplementary Information
