# Supplementary material for: Nanoscale characterization of PM2.5 airborne pollutants reveals high adhesiveness and aggregation capability of soot particles
Source: Sci Rep. 2015 Jul 16;5:11232. doi: 10.1038/srep11232 (PMC4503936; doi:10.1038/srep11232)
Supplement: Supplementary Information [file srep11232-s1.doc]

**Nanoscale characterization of PM2.5 airborne pollutants reveals high adhesiveness and aggregation capability of soot particles**

Yuanyuan Shi^1^, Yanfeng Ji^1^, Sun Hui^2^, Fei Hui^1^, Jianchen Hu^1^, Yaxi Wu^3^, Jianlong Fang^3^,Hao Lin^4^, Jianxiang Wang ^2^, Huiling Duan^2^*, Mario Lanza^1^*

^1^Institute of Functional Nano & Soft Materials, Soochow University, 199 Ren-Ai Road, Suzhou Industrial Park, Suzhou, Jiangsu 215123, China

^2^State Key Laboratory for Turbulence and Complex System, CAPT, Department of Mechanics and Engineering Science, College of Engineering, Peking University, Beijing 100871, China

^3^Chinese Center For Disease Control and Prevention, Institute of Environmental Heath and Related Product Safety,Beijing 100021,China

^4^Department of Mechanical and Aerospace Engineering, Rutgers, The State University of New Jersey, Piscataway, NJ 08854, USA

*Corresponding author: mlanza@suda.edu.cn, hlduan@pku.edu.cn

Index

PM_2.5_ collection process 2

Digital camera pictures of the contaminated filters 2

Optical microscope pictures of the contaminated filters 3

SEM-EDAX study of most representative particles on the filters 3

Methodology to select particles with rough and flat surfaces from SEM images 6

Silicon substrates used in this investigation 7

Process to transfer PM_2.5_ from rough filters to flat analysis-friendly substrates 7

Representative SEM images of PM_2.5_ transferred on Silicon 9

Universal groups of PM2.5 airborne pollutants 10

Reliability of the transfer method 13

Representative AFM images of PM_2.5_ transferred on Silicon 15

Correlation between AFM and SEM images 16

Quantification of the particles roughness with AFM 17

Statistical analysis of PM_2.5_ size transferred on Silicon 19

Analysis of PM_2.5_ deformation from topographic AFM maps 20

Values of adhesion force in reference substrates 21

Particle adhesion to the AFM tip 23

Measurement of the adhesion and deformation with AFM 24

Ability of Carbon-rich soot to aggregate other particles 25

Effect of some of PM_2.5_ to surrounding areas 30

**PM_2.5_ collection process**

PM_2.5_ airborne pollutants have been collected according to the regulations established by the US Environmental Protection Agency, Code of Federal Regulations 40, Part 53: "Ambient air monitoring reference and equivalent methods". For more information, please check: http://www.ecfr.gov. Mass concentrations of daily PM_2.5_ were acquired from the continuous monitoring using a TH-150C Automatic Medium Volume TSP Sampler (Wuhan Tianhong instruments Co. Ltd) with TH-PM_2.5_. Both equipments were located at the stepped platform in front of the laboratory building at No. 7 PanjiayuanNali road, in the Chaoyang District of Beijing (zip code 100021), in China. The particles were collected on December 2^nd^ and 7^th^, with concentrations of 106 µg/m^3^ and 298 µg/m^3^, respectively. We analyzed more than 30 samples and systematically detected three groups of universal particles: fluffy soot aggregate, elongated minerals and spherical fly ash (see Figures S10-S12). These three groups have been previously reported by many other authors at different locations of the world, including US, UK, Mexico, India, Italy and China (see Figures S10-S12). Therefore, the particles reported in this study are representative of the global collective of PM_2.5_ worldwide, and the aim of this work is not to further report their size/shape, amount and composition, but to thoroughly analyze the nanoscale properties of these three well-identified groups of PM_2.5_airborne pollutants.

**Digital camera pictures of the filters**


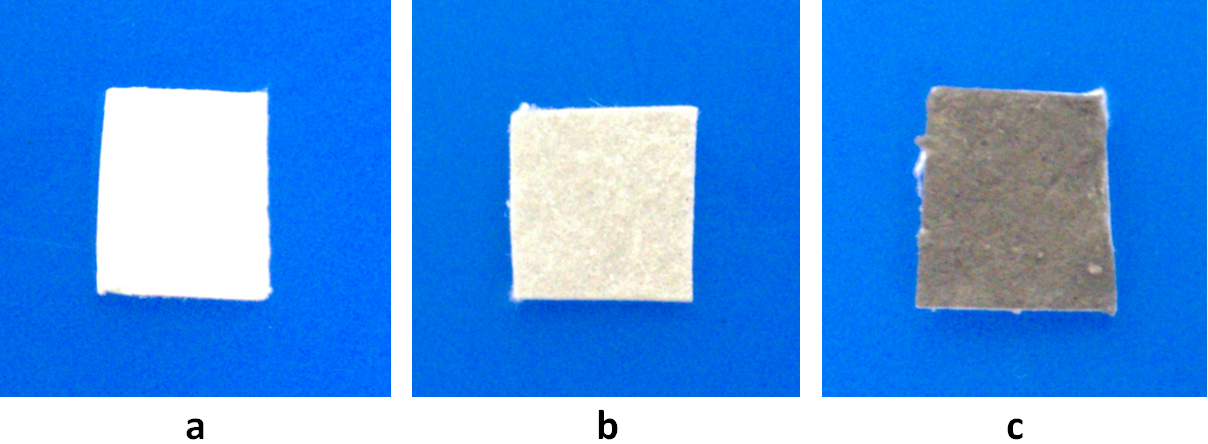


Figure S1: Pictures collected with a digital camera for the clean filter (a), and the particles exposed to atmospheric air on December 2^nd^ (b) and December 7^th^(c). Sizes: 1 cm x 1 cm.

**Optical microscope pictures of the contaminated filters**


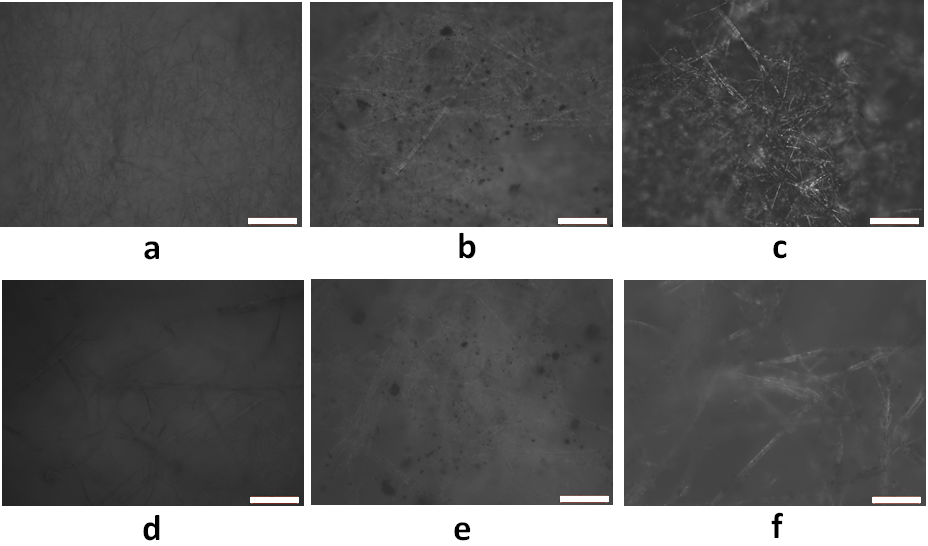


Figure S2: Optical microscope collected on clean filter ((a) and (d)), intermediate polluted filter ((b) and (e)), and very polluted filter ((c) and (f)). Polluted filters show larger amount of particles (black spots). The scale bars are 100 µm for images (a-c) and 20 µm for images (d-f).

**SEM-EDAX study of most representative particles on the filters**

**
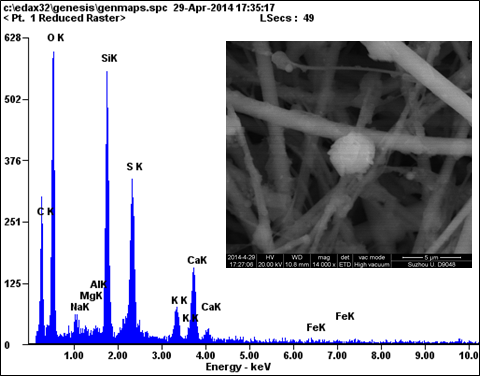
**

**Spherical fly ash**

Figure S3: SEM picture and EDAX analysis for one representative PM_2.5_ particle on the filter.


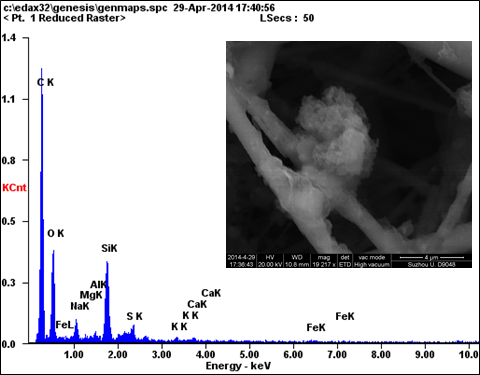


**Fluffy soot aggregate**


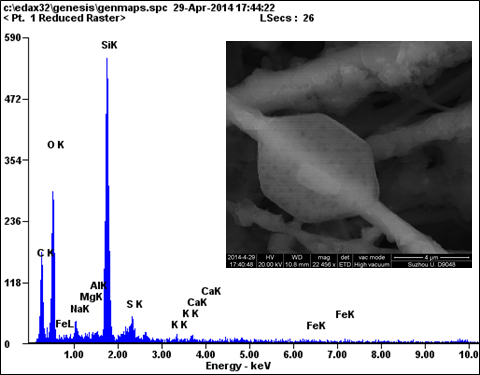


***Other* particle**

Figure S4: SEM pictures and EDAX analyses for two representative PM_2.5_ particles on the filter.


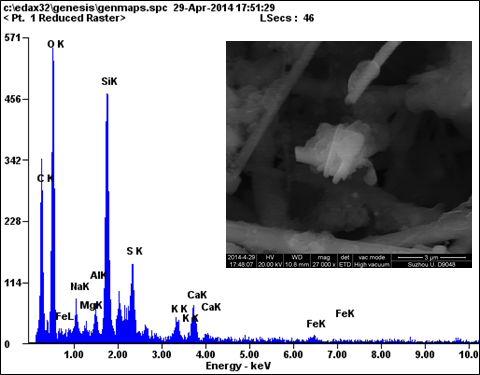


**Cluster of elongated minerals**


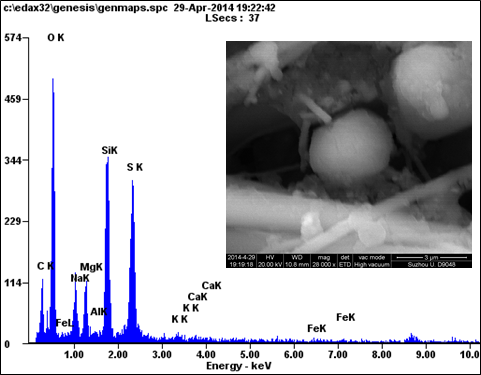


**Spherical fly ash**

**Elongated mineral**

Figure S5: SEM pictures and EDAX analyses for two representative PM_2.5_ particles on the filter.

**Methodology to select particles with rough and flat surfaces from SEM images**

According to the user manual from FEI [RS1], the SEM pictures collected by the Quanta 200FEG tool (used in this investigation) result from interactions between an electron beam with the secondary electrons of the atoms at or near the surface of the sample. Due to the very narrow electron beam, SEM micrographs have a large [depth of field](http://en.wikipedia.org/wiki/Depth_of_field), which allows displaying the features of a sample at different heights. Such topographic differences are displayed with different values of the grayscale in JPG files recorded. Therefore, the SEM images can also reveal information about the roughness of the samples.


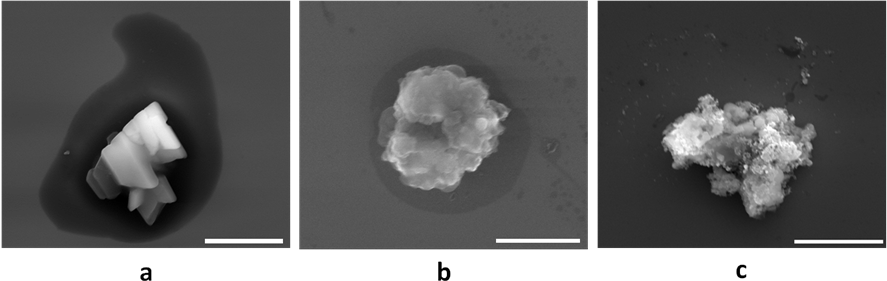


Figure S6: Representative PM_2.5_ particles with flat (a), semi-rough (b), and rough (c) surfaces. The scale bars are 4 µm for (a), 1 µm for (b) and 4µm for (c).

Moreover, most of the particles scanned with SEM can also be found with the AFM tip. After transferring the particles on the grid-like SiO2/Si substrate, we perform SEM images of the samples to find out the PM_2.5_. Then, we zoom out until a global picture of the whole sample at macroscopic scale is obtained. Finally, we go to the AFM and locate the tip at the same place and perform a large area scan. Despite the shape of some PM_2.5_ could be slightly modified during the scan, we found relatively easy most of the particles analyzed with EDAX/SEM in tapping mode. In contact mode it was more difficult because the AFM tip may induce particle shape modification, especially in those with less stable shapes. SEM/AFM corrlation is also possible on bare Silicon depending on the particle size and shape. The AFM then can quantitatively compare the differences on the surface roughness of the particles by using both the roughness analysis and cross section tools. Please see the section "Correlation between AFM and SEM images" of this supplementary information document for more details.

**Silicon substrates used in this investigation**


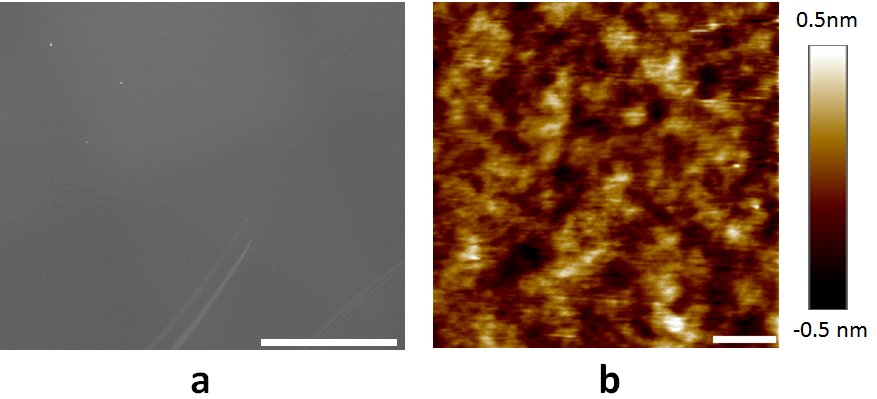


**RMS = 198 pm**

Figure S7:SEM (a) and AFM (b) images of clean Si. For this investigation, a n-type Si with natural oxide layer from Kejing Co. Ltd. was used. Before PM_2.5_ transfer, the wafers were cleaned in isopropyl alcohol (AR) in ultrasonic bath for 5 minutes and then rinsed in pure water during 10 seconds (without sonication). The samples were dried with dry nitrogen gas. Nearly no particles exist on the surface of clean Si. The scale bars are 500µm for (a) and 1.6µmfor (b).The roughness of the Si substrate is very low and suitable for particle detection.

**Process to transfer PM2.5 from rough filters to flat analysis-friendly substrates**

This is a method commonly used in chemistry to homogenize solutions, and it basically introduces mechanical stresses in the sonicated samples. According to the Royal Society of Chemistry (ref. 31 in the manuscript), ultrasonication can be defined as: the irradiation of a liquid sample with ultrasonic (>20 kHz) waves resulting in agitation. Sound waves propagate into the liquid media result in alternating high-pressure (compression) and low-pressure (rarefaction) cycles. During rarefaction, high-intensity sonic waves create small vacuum bubbles or voids in the liquid, which then collapse violently (cavitation) during compression, creating very high local temperatures. In our case, we sonicate the samples in the absence of any solvent. As shown in Figure S8, we used no water or any other solvent in the tube (between the Silicon substrate and filter). On the other hand, both the glass and sonicator contained water. Such configuration produced the continuous vibration of both the tube and the sample, but no aggressive reaction took place on the PM_2.5_ surface. As a result, the particles can detach from the filer and precipitate on the target substrate, but no remarkable particles size change or fragmentation has been observed in further SEM images. It is worth noting that the density of particles (number per mm^2^) is not representative (it cannot be controlled), since the mechanical stress and filter/Silicon distance may not be homogeneous during the transfer process, and we didn't find any characteristic value of particle density.


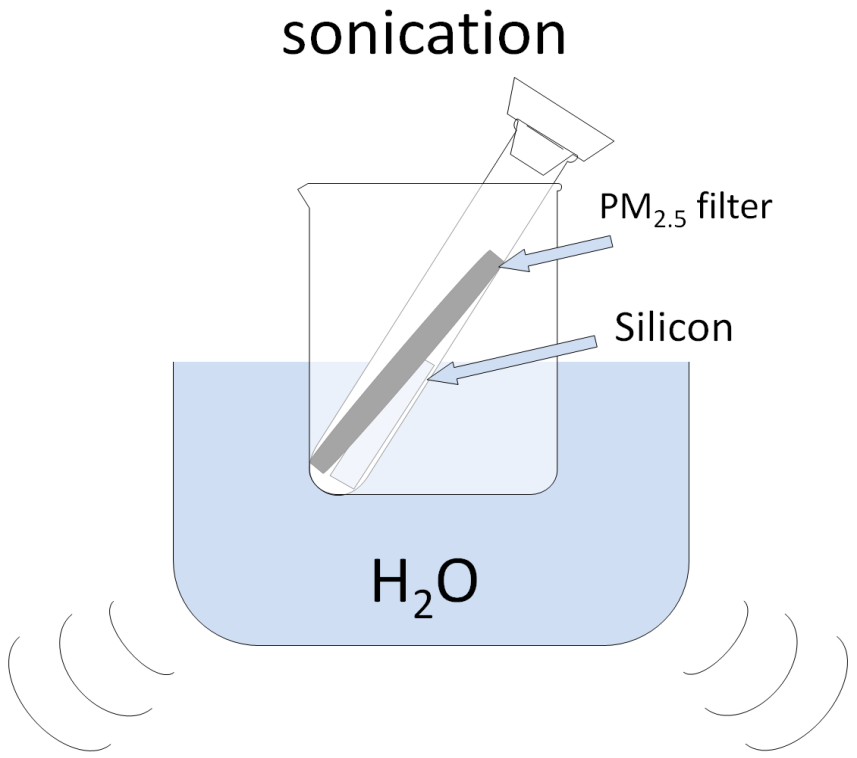


Figure S8: Schematic of process followed to transfer PM_2.5_ from rough filamentary filters to clean Si. A piece of polluted filter and cleaned Si are introduced in a plastic tube box (free of any solvent). Then, the tube is placed in a glass with water and introduced into the ultrasonic bath for different times. By this method, the PM_2.5_ can be effectively transferred to a flat substrate avoiding particle modification.

**Representative SEM images of PM_2.5_ transferred on Silicon**


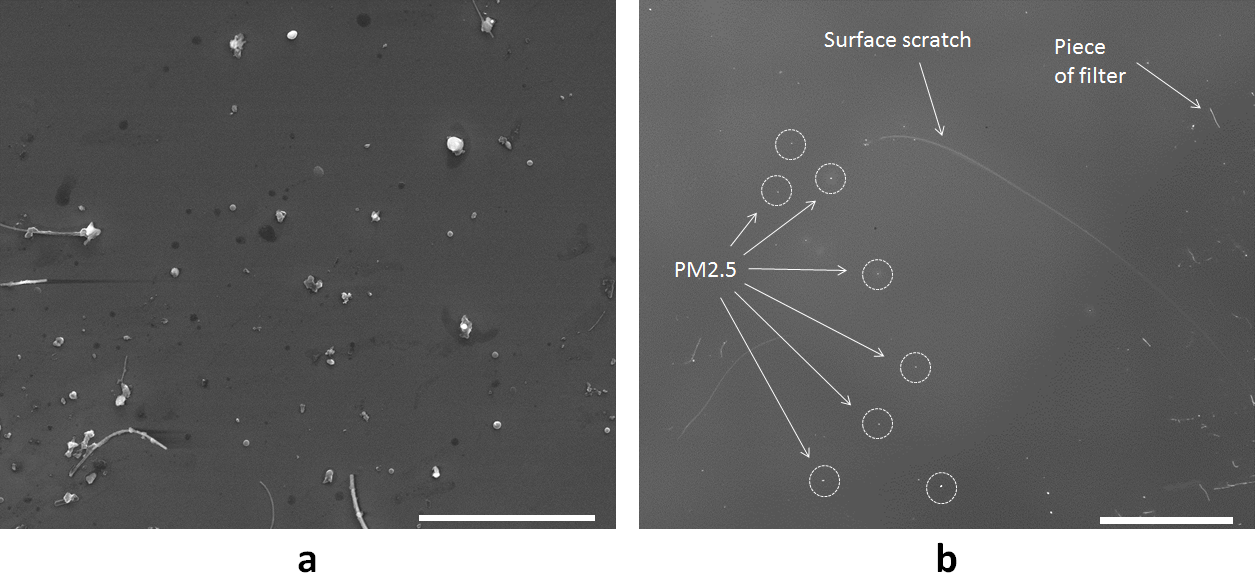


Figure S9: Large area SEM images of the particles on clean Si after the transferring process described above. The PM_2.5_ particles, pieces of filters and surface scratch can be clearly observed on the surface of clean Si. The whitish dots in (a) and (b) are the PM_2.5_ particles. The short filaments are parts of filters, and the long trace in (b) is just the surface scratch. The scale bars are 40 µm for (a) and 500 µm for (b).

**Universal groups of PM_2.5_ airborne pollutants: carbon-rich fluffy soot aggregate**


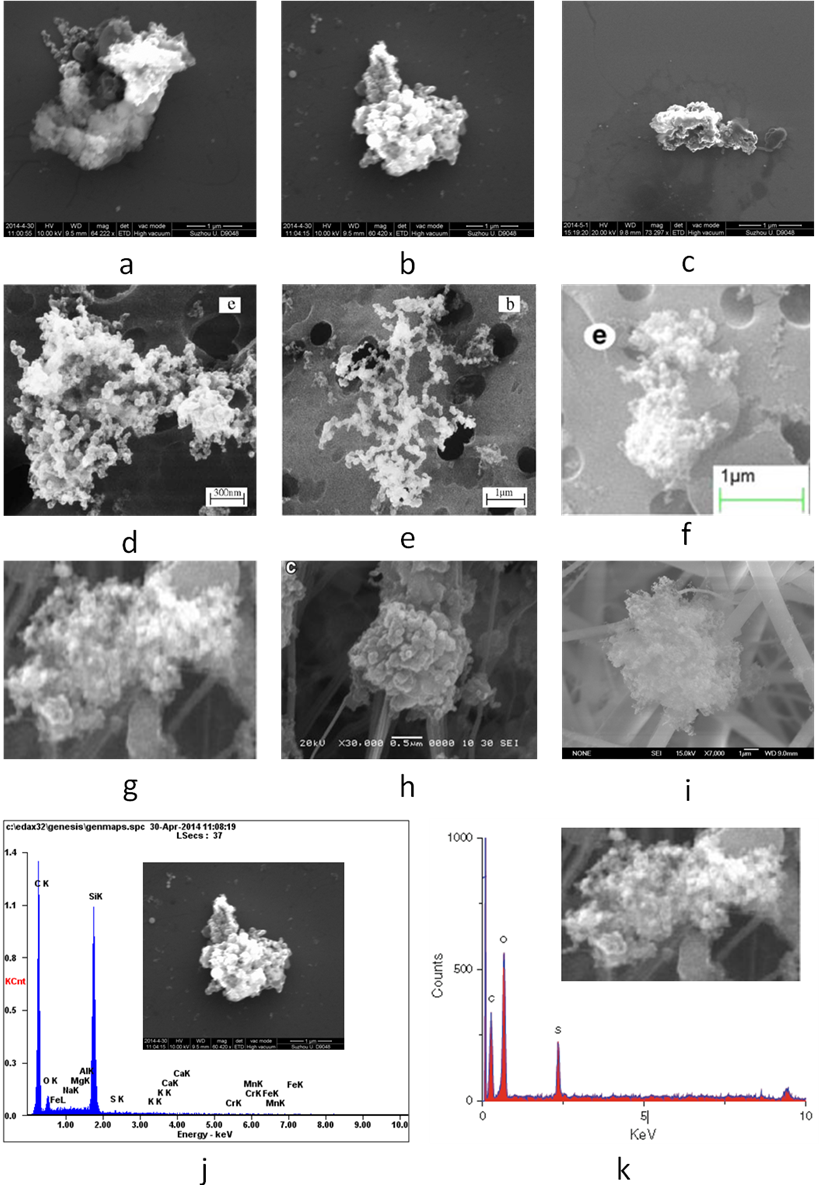


Figure S10. SEM images of soot aggregate. (a-c) and (j) are data from this investigation (Beijing, China). (d) and (e) are from reference [3] (Shanghai, China). [f] is from reference [5] (Guangzhou, China). (g-h) and (k) are from reference [19] (Pune, India). (i) is from reference [RS5] (Wellington, New Zealand).The chemical composition in (j) and (k) by EDAX.

**Universal groups of PM_2.5_ airborne pollutants: elongated minerals rich in metal-silicates**

**
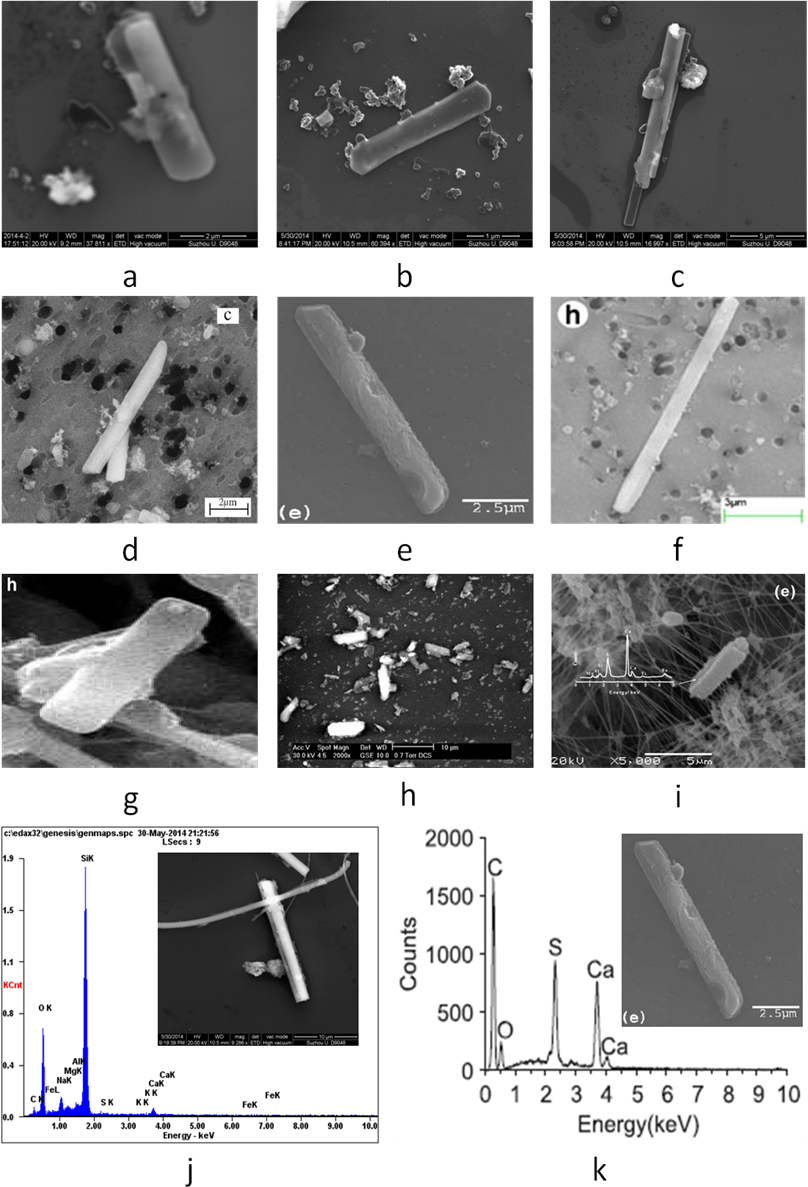
**

Figure S11: SEM images of elongated minerals. (a-c) and (j) are data from this investigation. (d), (f) and (g) are from references [3] (Shanghai, China), [5] (Guangzhou, China)and [19] (Pune, India), respectively. (e) and (k) are from reference [18] (all aroundUSA). (h) is from reference [RS6] (Gujarat, India). (i) is from [RS7] (Tianshan, China).

**Universal groups of PM_2.5_ airborne pollutants: spherical fly ash rich in metals**


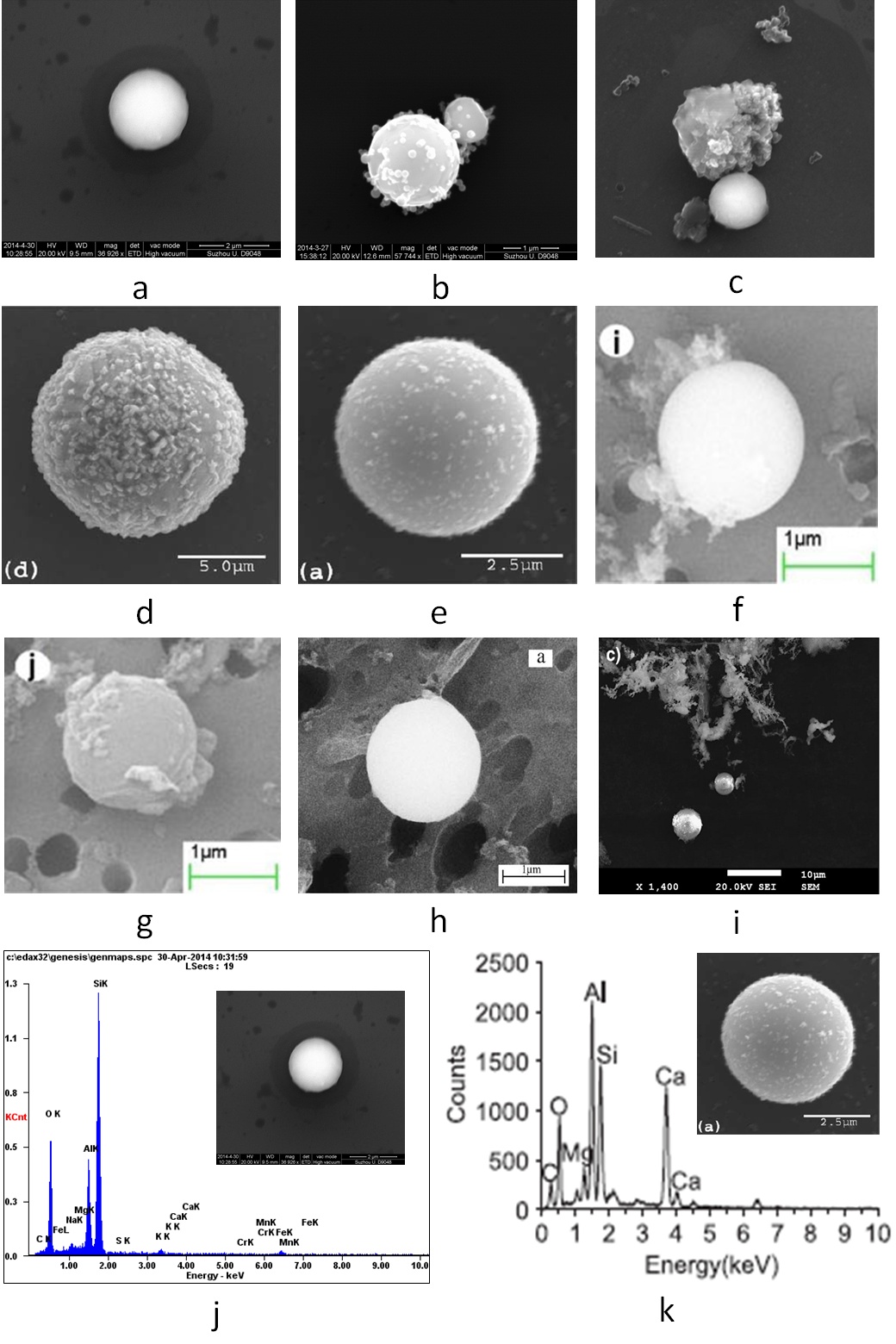


Figure S12. SEM images of fly ash. (a-c) and (j) are data from this investigation. (d), (e) and (k) are form reference [18] (all around USA). (f) and (g) are from reference [5] (Guangzhou, China). (h) is from reference [3] (Shanghai, China). (i) is from reference [RS8] (Lisbon, Portugal).The scale bars for (b) and (c) are 3 µm.

**Reliability of the transfer method**

In order to analyze the reliability of the transfer method we performed an additional experiment, which consisted on attaching a piece of Silicon on the filters before introducing them in the PM_2.5_ collector (Figure S13a). We in-situ collected the PM_2.5_ particles on the Silicon substrate, and we observed many particles with sizes, shapes and compositions very similar to those reported after the transfer (Figure S13b-f and S10-12). This is indeed demonstrating that the transfer process didn't dramatically alter the properties of the particles we reported in the manuscript.


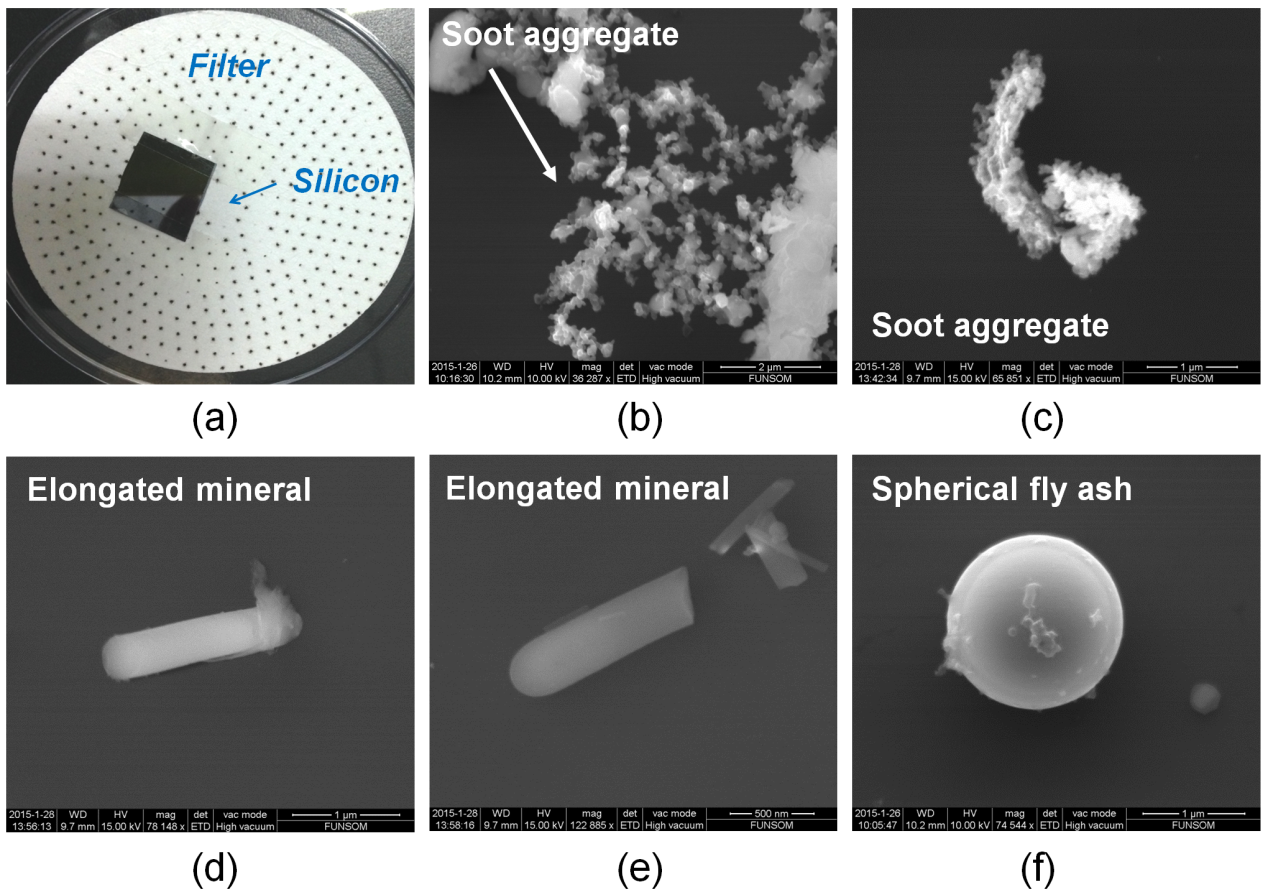


Figure S13: (a) Filter with a piece of Silicon in the center. This filter was directly introduced in the collector for in-situ studies. (b-f) In-situ collected particles on the Silicon substrate. The particles collected are very similar to those observed in the manuscript after transfer.

It is worth noting that after a carefully analysis of the particles, we observed that the density of small particles (diameter below 500 nm) attached to the Silicon remarkably increased when using in-situ collection (Figure S14a). This indicates that some smaller particles attached in the filter couldn't be transferred on the Silicon during ultrasonication. Probably, due to their smaller mass, the detaching force was smaller than the adhesion force to the filter, and therefore such small particles just remained on the filter. Finally, some atmospheric particles can be semi-to-entirely liquid when they are airborne, which would difficult the filter-to-Silicon transfer process. Interestingly, many particles collected in both in-situ (Figure S14d) and transferred (Figure S28) experiments show a dark trace that, due to its regular contour and flat shape, may be related to the semi-liquid nature of some particles


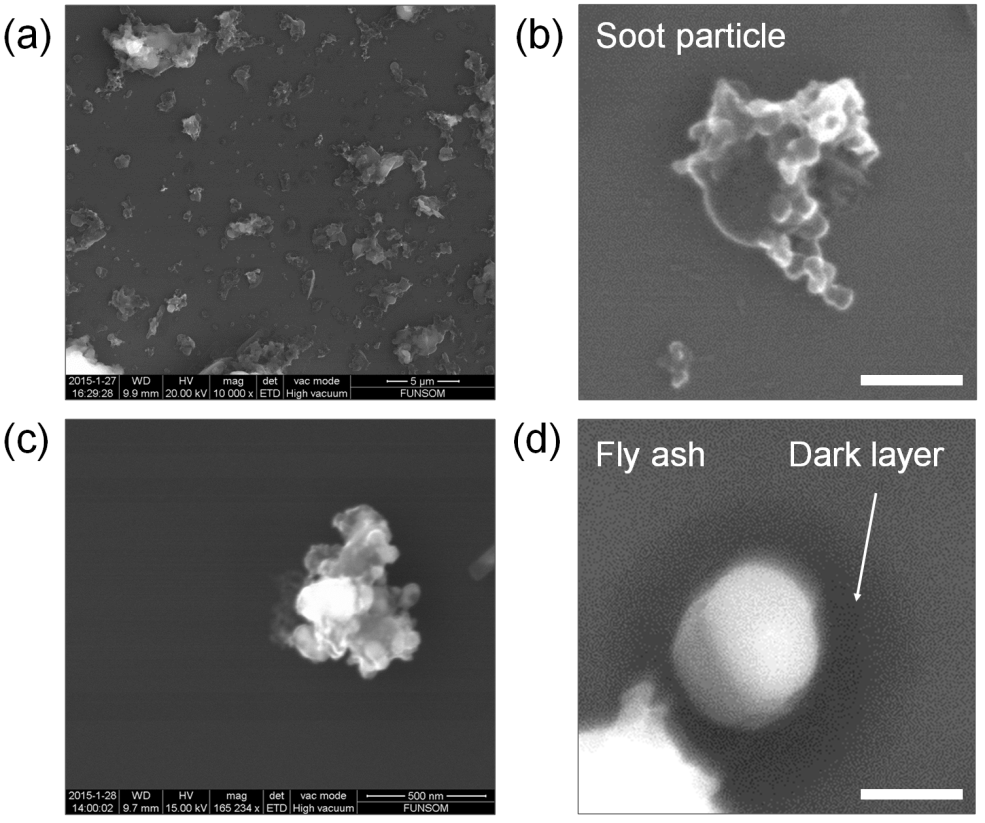


Figure S14: (a-b) SEM image of the in-situ collected particles on the Silicon filters. Unlike after particle transfer, a large density of small particles has been observed on the Silicon. (b-c) Examples of small size particles showing that their nature is very similar to the larger ones, indicating that they may be related to small portion of those reported in the manuscript, rather than a different type of particles. (d) Example of a particle surrounded by a dark layer that may be related to liquid particles. The scale bars in (b) and (c) are 250 nm and 200 nm respectively.

We careful analyzed the morphology of these small particles by SEM and observed that their morphology and composition is very similar to that of larger particles (Figures S14b-c and Figures S10-12), indicating that they may not necessarily correspond to a new type of particles, but they could be just small fragments of soot. We would like to highlight that, using in-situ collection on Silicon, we observe a larger density of particles surrounded by a darker layer (as shown in Figure S14). The long and straight shapes of this surrounding layer make us think that this could be related to the semi-liquid particles.

**Representative AFM images of PM_2.5_ transferred on Silicon**


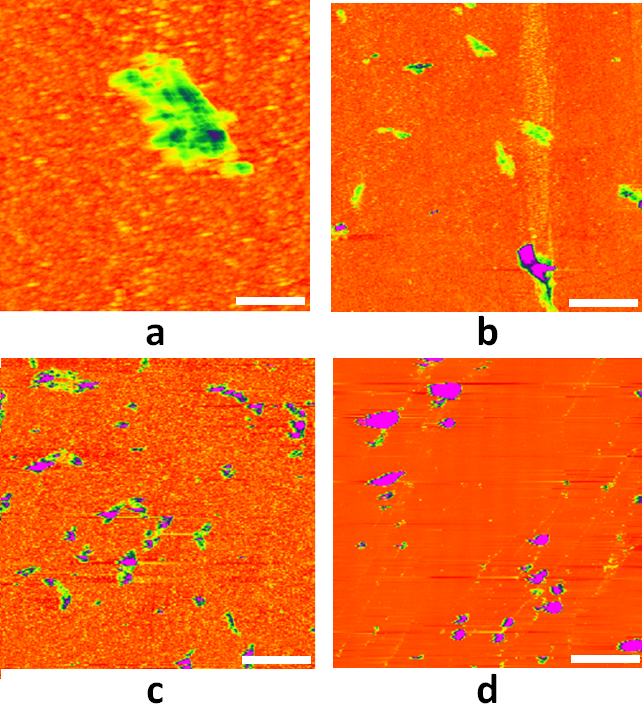


Figure S15: Topographic AFM images displaying different types of PM_2.5_. The particles in figures (a) and (b) have a 2 nm Au coating (a-b), while (c) and (d) were measured as-transferred. In the paper, all topographic images were displayed without gold coating. The substrate used in figures (a-c) was glass, while figure (d) used Si. Many PM_2.5_particles can be observed in all the AFM images and they usually exhibit different shapes. The AFM used in these experiments was the Veeco Multimode V AFM from Bruker in tapping mode. For tapping measurements Si tips from Nanoworld(model NCH-20) were used. We used a scan line frequency of 1 Hz and the amplitude setpoint ranged between 100-300 mV. The scale bars are 1µmfor image (a), 5 µm for image (b), 10 µm for image (c) and 4 µm for image (d).

**Correlation between AFM and SEM images**

**
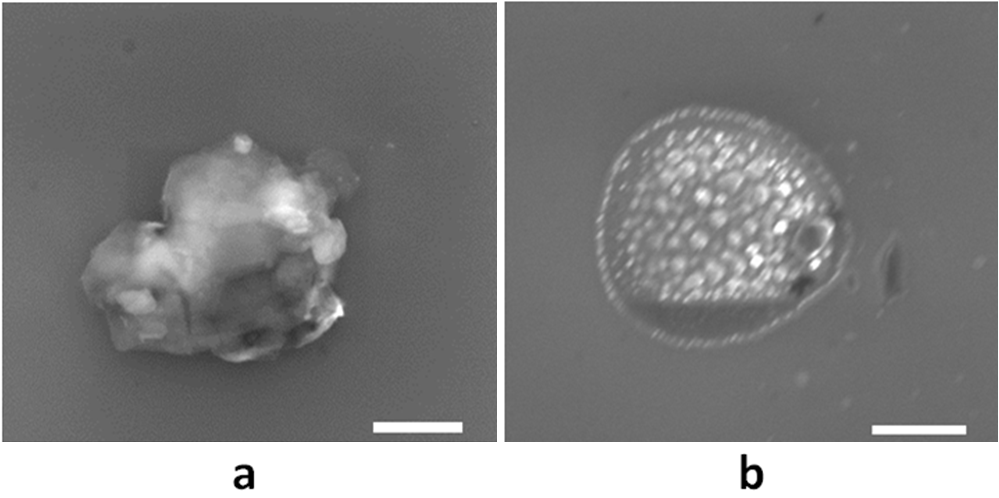
**

Figure S16:(a) and (b) show the SEM images correlated to the three dimensional AFM topographic maps in Figures 2a and 2b of the article (respectively). Most of the particles can be found with the tip of the AFM after SEM and vice versa. The scale bars are 1 µm for (a) and (b).

**Quantification of the particles roughness with AFM**

Among other uses, the high lateral and vertical resolution of the AFM can be used to quantify differences on the surface roughness of the particles, so that they can be accurately classified. For this purpose, we use the Roughness tool of the AFM can be used to calculate the RMS value of an area selected. The layout of the AFM tool in the AFM software is shown in Figure S17. The *Rq* value corresponds to the standard deviation of the Z values within the box cursor, calculated as:


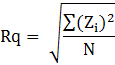


where *Zi* is the current Z value, and *N* is the number of points within the box cursor.


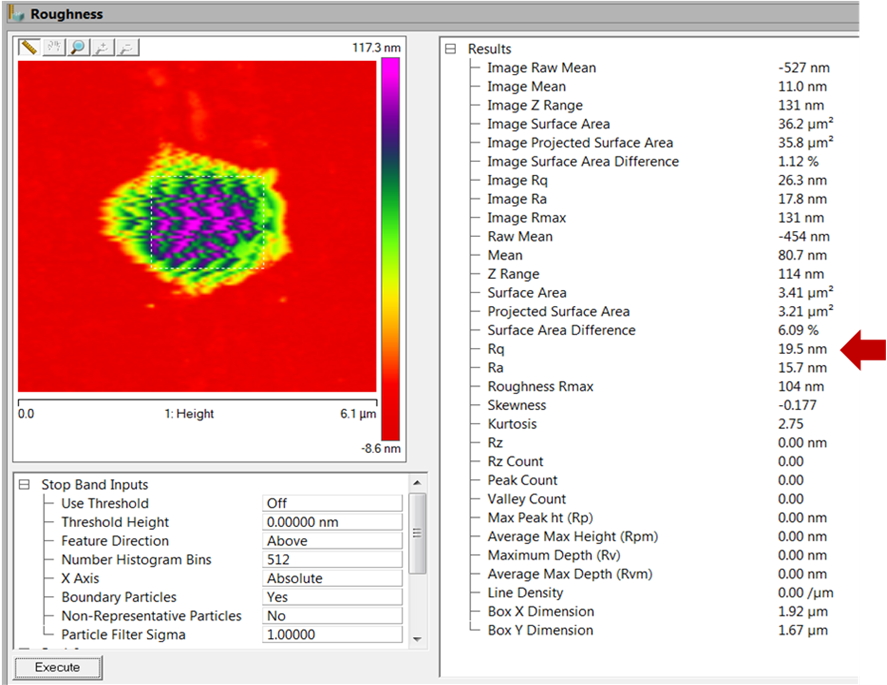


Figure S17: Layout of roughness analysis in Bruker Nanoscope Analysis Bruker version 1.40.

Additionally, we compare the roughness of the particles with the section tool. In this case, the software provides the standard deviation (*RMS*) of the Z values between the reference markers.


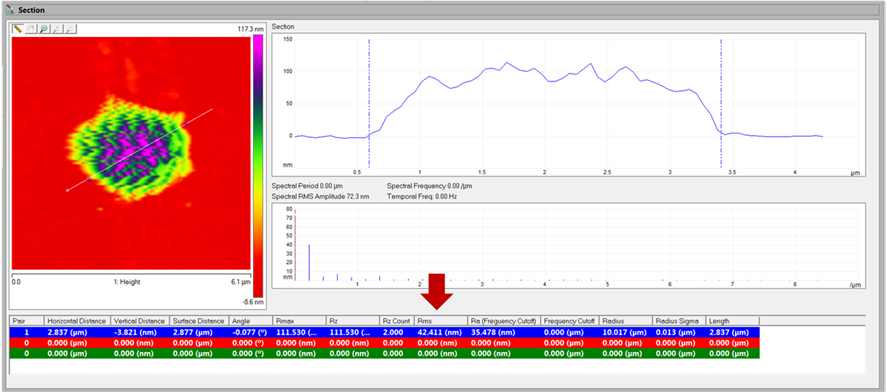


Figure S18: Layout of the cross-section tool in Bruker Nanoscope Analysis Bruker version 1.40.


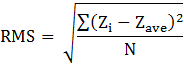


where*Zi* is the current Z value, *Zave* is the average of the Z values between the reference markers, and *N* is the number of points between the reference markers.

Despite we observe differences on the values calculated, the AFM software allows reliable comparison of the surface roughness of the particles. It is worth noting that the Rq value calculated with the roughness can change depending on the box size. For this reason, we used a constant box size of 500nm x 500 nm for all analyzed particles. The numerical data obtained with the software of the AFM correlate with the visual observations made from SEM images in 88.46 % of the particles analyzed.

**Statistical analysis of PM_2.5_size transferred on Silicon**


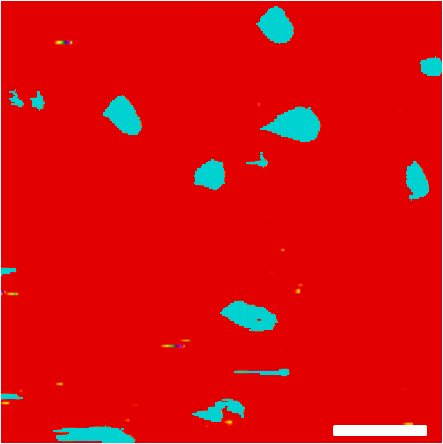


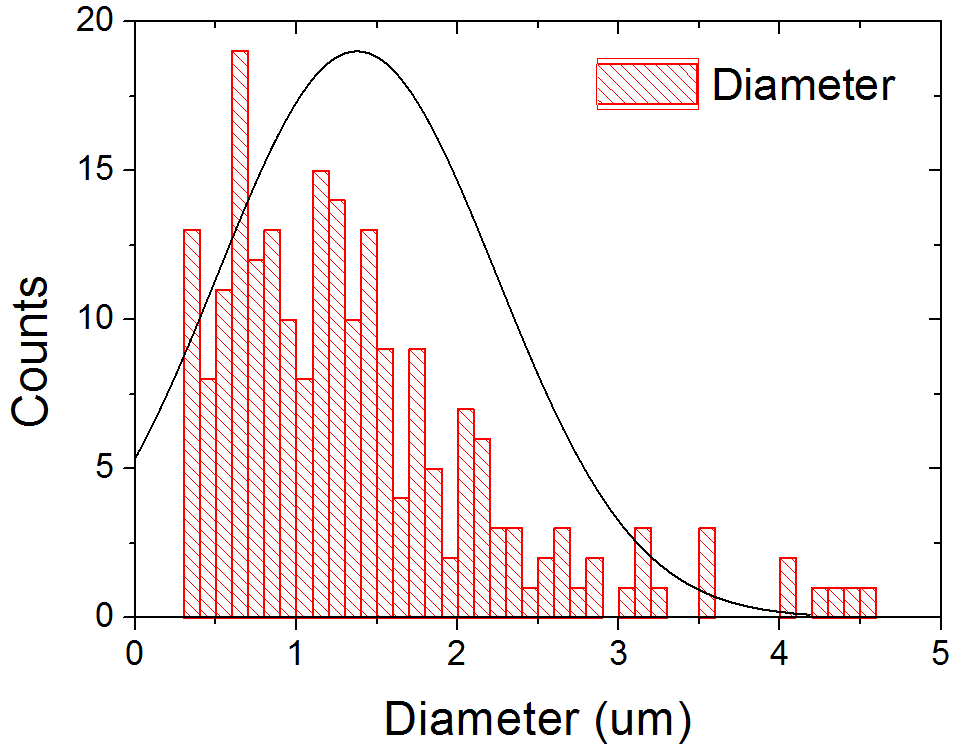

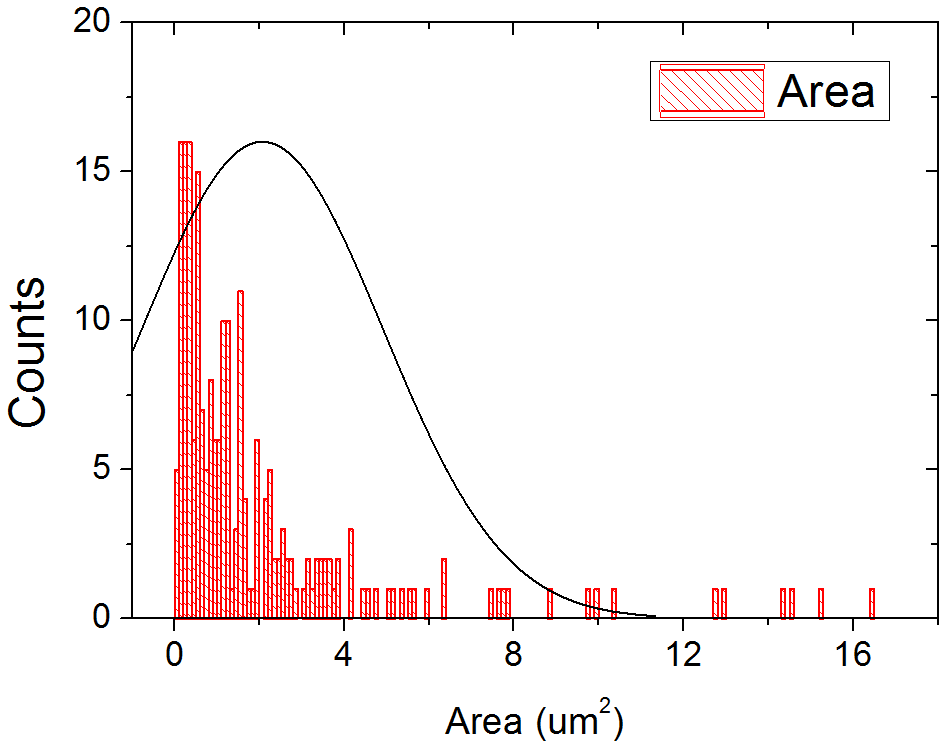


Figure S19: Statistical analysis of the physical diameter (b), and area (c) of PM_2.5_ particles transferred on clean Si. The AFM software, NanoScope Analysis V1.4 was used to do the particle analysis. As an example panel (a) shows the typical aspect of the processed images, with areas above a threshold height highlighted in blue. The threshold height was selected to be 10 nm above the most repeated value of the image histogram, which is represent the average height of the Silicon substrate. In total 217 particles in different images were analyzed. As it is shown in (b), the main diameters of the PM_2.5_ range from 0.4-2.2 µm, in agreement with the values reported in other works [RS2-4] and expected values (below 2.5 µm). As indicated in the manuscript, despite the values here displayed represent the physical size, the quadratic relationship between particle density and aerodynamic diameter (see Eq.1 of the manuscript), the values of PM_2.5_ densities previously reported and the fact that aggregates of particles show a much lower density than bulk materials allows considering that most of the particles in figure S19b are in fact PM_2.5_ airborne pollutants. The corresponding area-counts histogram further demonstrates the main size of these particles. The scale bar in (a) is 10 µm.

**Analysis of PM_2.5_ deformation from topographic AFM maps**


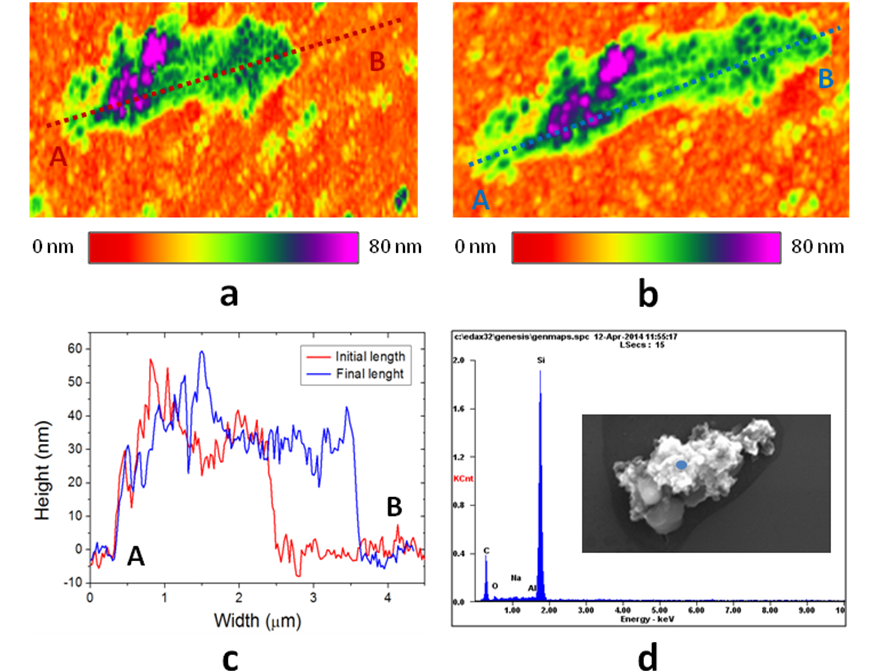


Figure S20: First (a) and fourth (b) topographic AFM images of PM_2.5_ particles transferred on clean Si. Both scale sizes are 5.5µm, and the drive amplitude used during each scan was 100 mV for (a) and 300 mV for (b). The drive amplitude is a parameter used when scanning in tapping mode and it is directly proportional to the tip/particle contact force. As it can be observed, at higher contact forces the particle can be elongated. It is worth noting that (for this particle) deformation has been achieved when applying a drive amplitude of 300 mV, while drive amplitudes of 100 mV and 200 mV didn't produce deformation. Shape modification can be also observed from the cross sections (c). Further topographic maps measured with high and low contact forces didn't show additional shape modificationrespect to (b), indicating that the particle modification was plastic (larger viscosity), and elastic particles that recover the initial shape have been rarely detected. The chemical composition analysis with EDAX (d) revealed that PM_2.5_ with such morphology and shows that this elastic particle contains a lot of carbon.

**Values of adhesion force in referent substrates**

To better understand the meaning of the adhesion force values measured on the PM_2.5_, we perform additional F-Z curves on four reference substrates: Silicon, Glass Aluminum plate for AFM samples and adhesive tapes: usual scotch tape and laboratory carbon tape.


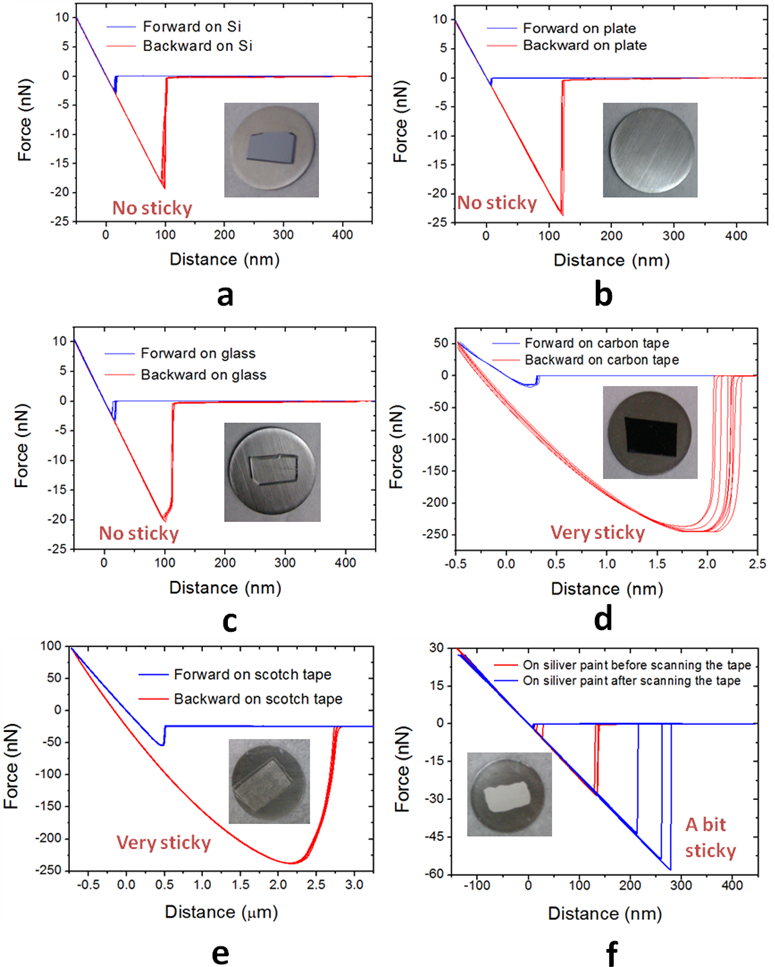


Figure S21: Force-Distance curves on four reference substrates with different stickiness: cleaned Si (a), Aluminum plate (b), cleaned glass (c) carbon tape (d), scotch tape (e). (f) F-Z curves on dried silver paint before and after the scotch tape experiments in (e). The large stickiness alters the properties of the AFM tip, the same behavior observed in some specific ultra-sticky particles.

The Force-Distance curves measured on Silicon revealed adhesion forces around -20 nN (figure S21a) consistent with values previously reported [RS9]. Similar experiments performed on AFM sample holders made of aluminum (b) and a glass slides (c) revealed values of the same order of magnitude than silicon. On the contrary when measuring Force-Distance curves on sticky carbon tape, adhesion forces more than ten times larger (around -230 nN) have been observed. The conventional scotch tape used also showed similar adhesion force values. Figure S21e shows the F-Z curves on a plate covered with dried silver paint before and after the scotch tape experiments. Therefore, the PM_2.5_ particles with adhesion values close to 20 nN could be considered non sticky, while those near 200 nN should be highlighted as very sticky.

**Particle adhesion to the AFM tip**


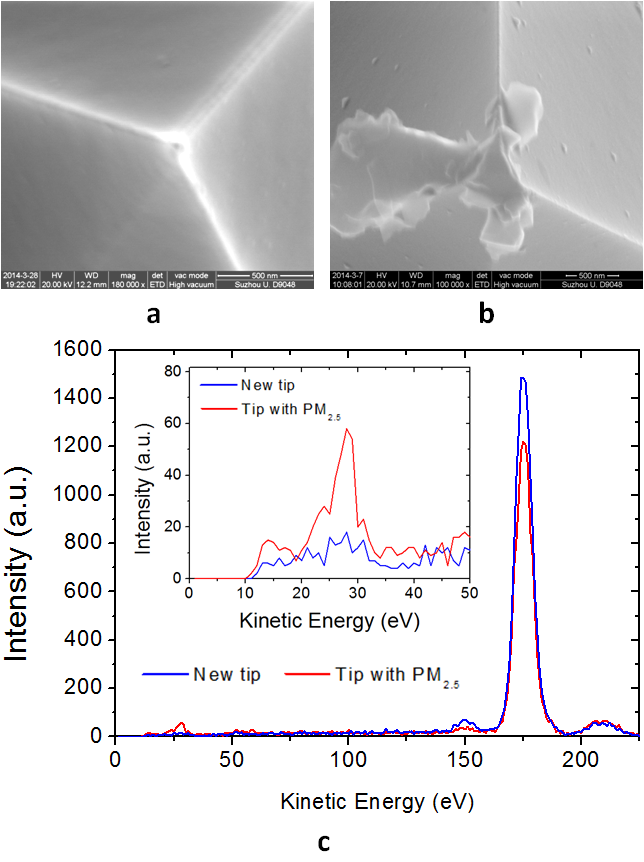


C

Si

Figure S22: SEM images of a Pt-Ir coated AFM tip before (a) and after (b) scanning very sticky PM_2.5_ particles on clean Si in contact mode. The EDAX survey measured on the tip apex of each image (c)is shown below. Something attached to the tip in (b), which shows similar chemical composition to the sticky PM_2.5_ particles in (c). This further demonstrates the adhesion property of PM_2.5_. The scale bars are 500 nm for (a-b).

**Measurement of the adhesion and deformation with AFM**

When analyzing the properties of small particles with the AFM, the shape of sequences of Force-Distance (F-Z) curves can provide information about electrostatic force, tip/particle adhesion, particle deformation and particle rupture and [29].The Force-Distance curves were recorded with the Multimode AFM working in contact mode using a Pt-Ir coated AFM tip from Bruker. The tips we used were standard commercially available AFM tips from Bruker (model SCM-PIC). These tips were made by silicon micromachining and were coated first with a 20 nm thick layer of Pt–Ir, which is a 95% platinum and 5% iridium alloy (the iridium is used to enhance the stability of the platinum layer). The other main characteristics of the tip are: thickness = 2 µm, width = 50 µm, length =450 µm, spring constant =0.2 N/m, resonance frequency = 13 kHz and nominal tip radius =20 nm. More sophisticated AFM allow analyzing the tip-sample interaction in real time during an scan in tapping mode. Using the Dimension Icon AFM from Bruker we have access to the Peak Force Quantitative Nanomechanical Mapping (QNM) tool. Using this mode particle adhesion and deformation maps can be built from the information collected at each point (pixel) during the scan (see Figure 3). During this mode, we usedCo-Cr coated silicon tips from Burker. The main characteristics of the tip are: thickness = 1.85 µm, width = 30 µm, length = 125 µm, spring constant = 5 N/m, resonance frequency = 150 kHz and nominal tip radius = 35 nm. The maps were recorded using a scan frequency of 1 Hz and a drive amplitude of 5V.


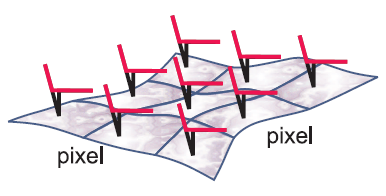


Figure S23: Schematic representation of the Peak Force QNM tool of the Dimension Icon AFM. Reproduced from [29]. Copyright @ 2013, by Jelena Zivkovic.

**Ability of Carbon-rich soot to aggregate other particles**

The fluffy Carbon-rich soot aggregate (which is sticky) can retain other species


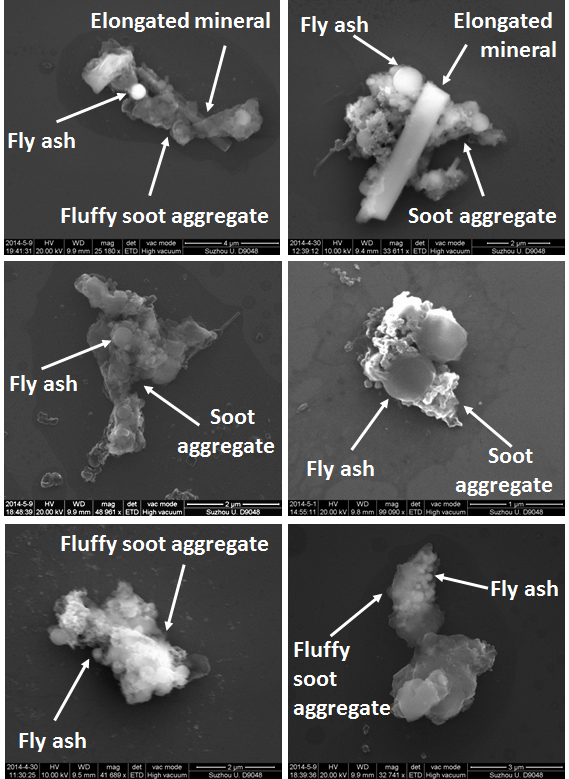


Figure S24: Examples of PM_2.5_ aggregate surrounded by a fluffy soot aggregate network.

We confirm the composition of the sticky network formed by the fluffy soot aggregate particles by means of EDAX. As an example, the images below show an aggregate of fly ash circular particles made of aluminum and iron oxides. The network of particles is aggregated by a continuous mass of fluffy Carbon -rich soot particles.

**
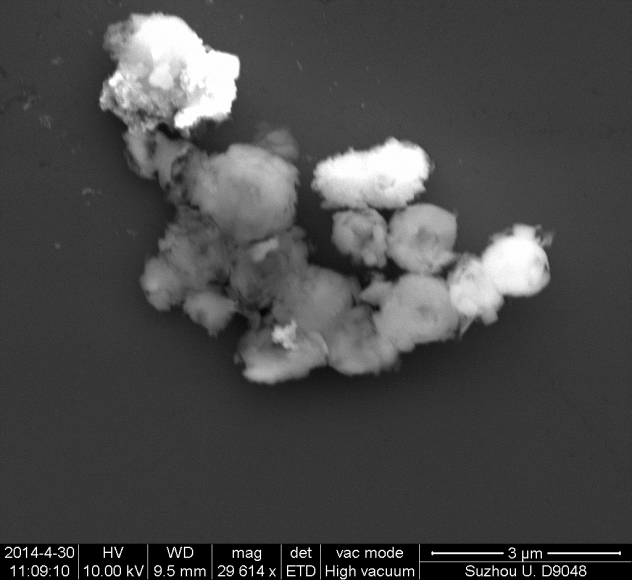
**

**Many fly ash aggregated by fluffy soot aggregate**

**
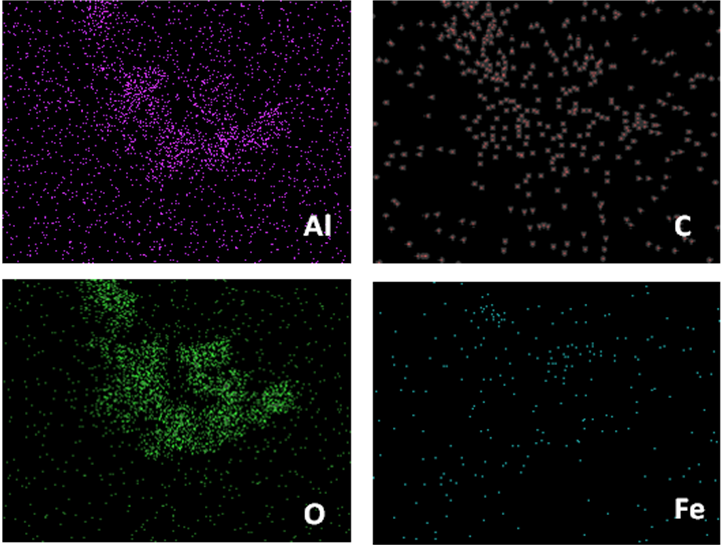
**

**
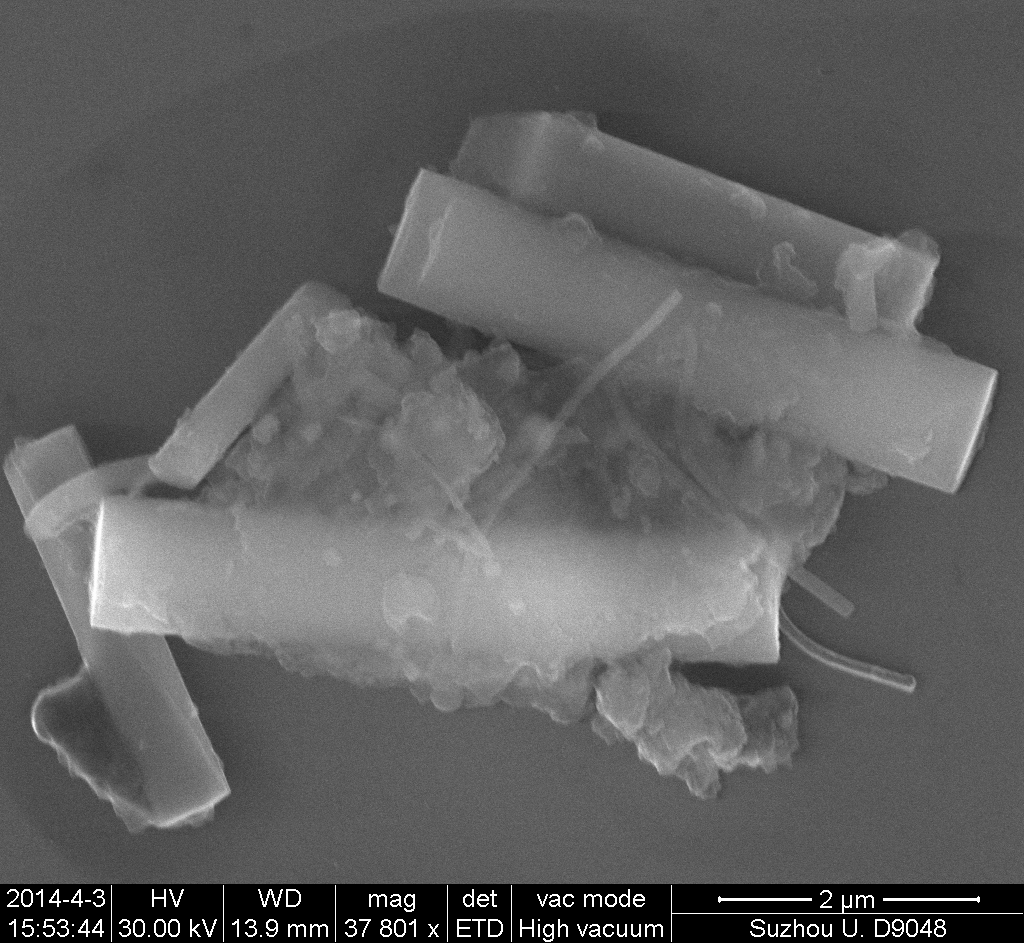
**

**Elongated mineral**

**Soot aggregate network**


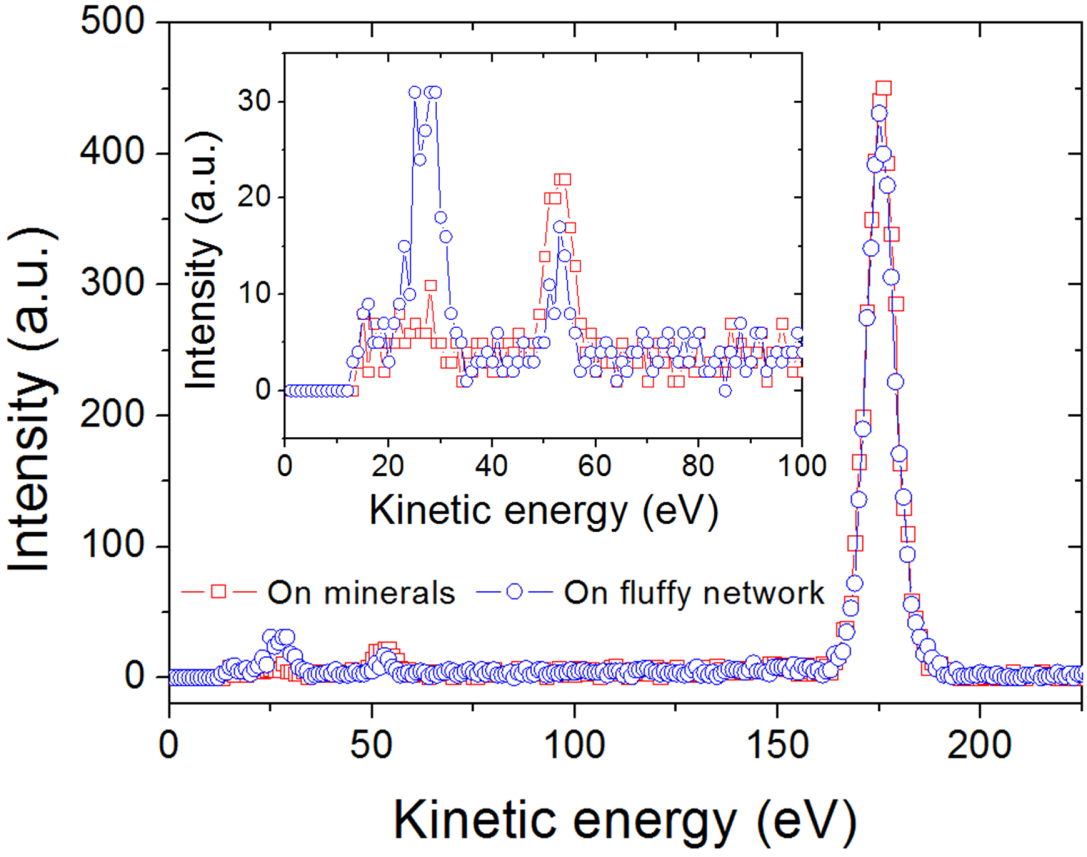


C

O

Si

Figure S25: Another example of PM_2.5_ aggregate. The top SEM and bottom EDAX images show a particle cluster and its chemical composition. The elongated mineral and fly ash get trapped in the Carbon-Oxigen rich network.

**
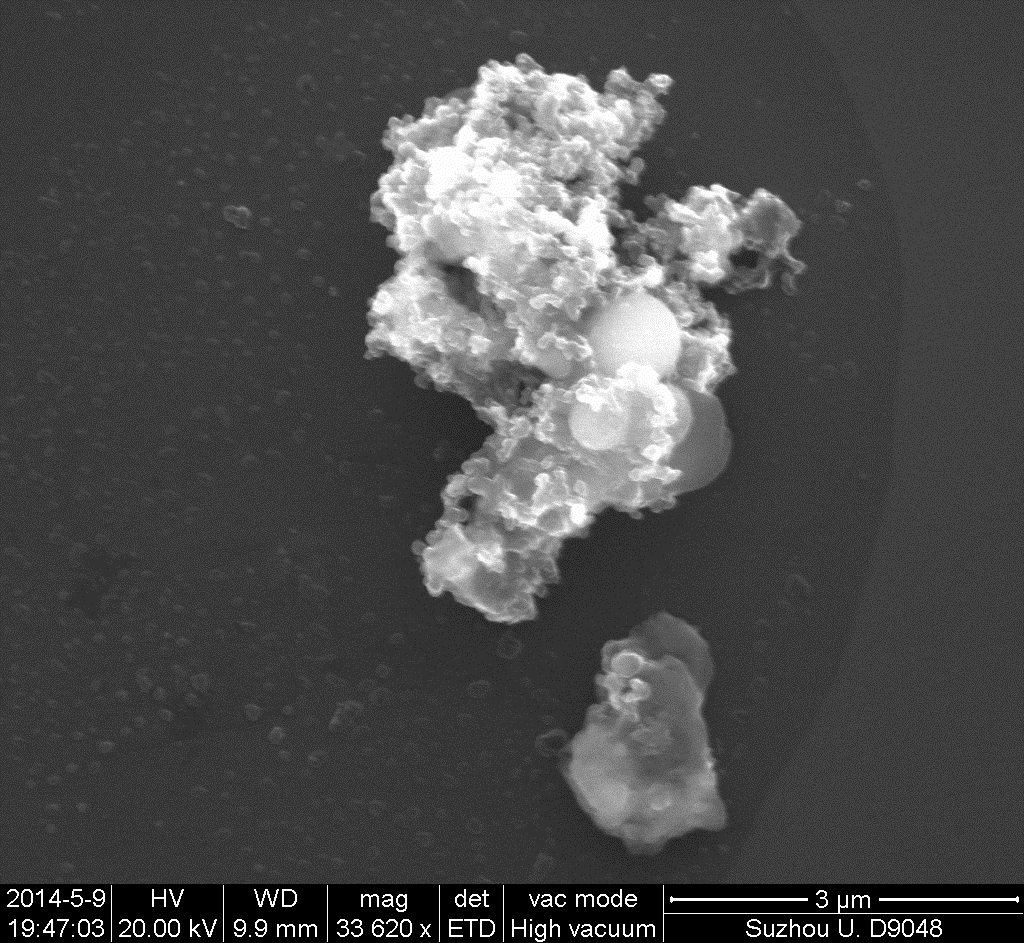
**

**Fly ash**

**Soot aggregate**

**
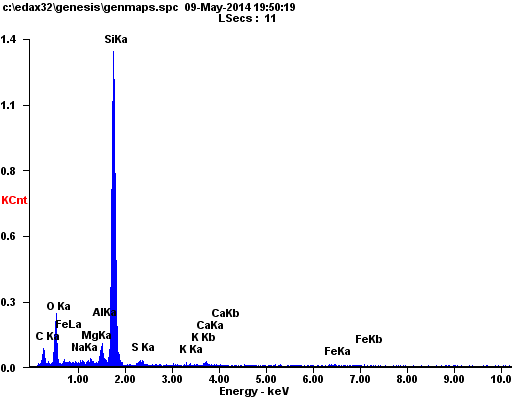

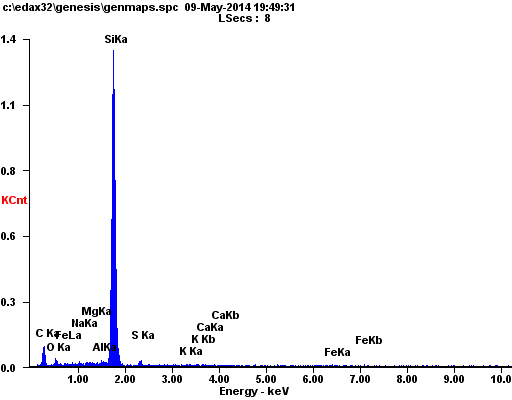
**

Figure S26: Another example of PM_2.5_ aggregate.The EDAX analyses show a larger concentration of Sulfur and Oxygen on the fly ash, while the Carbon peak is larger on the soot aggregate.


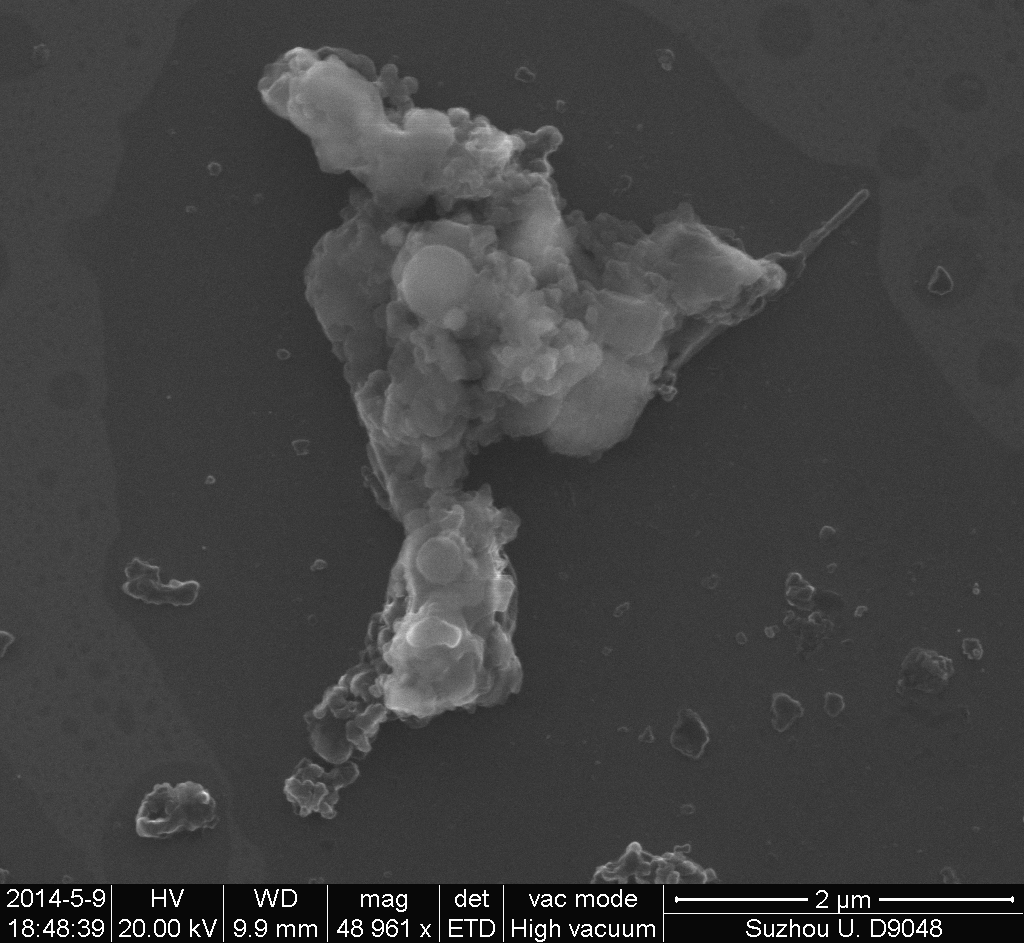


**Fly ash**

**Soot aggregate**

**
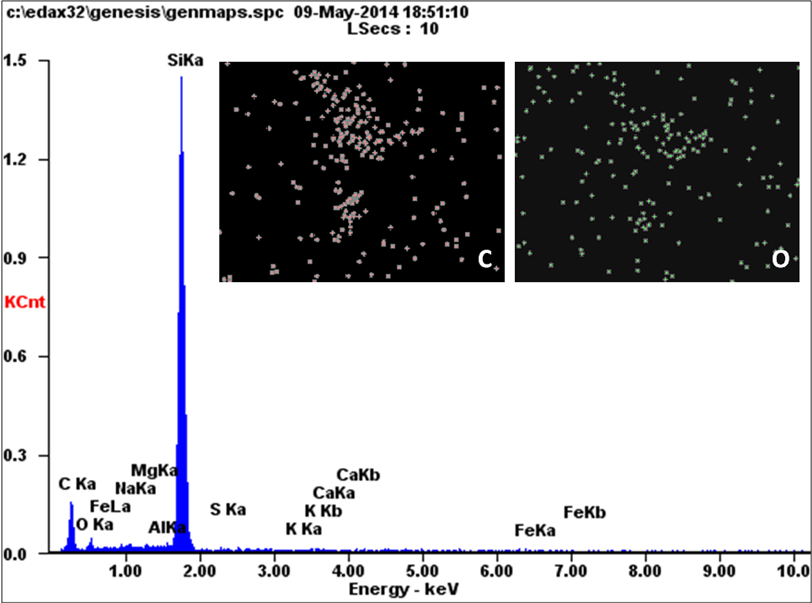
**

The carbon network aggregate

the rest of particles

Figure S27: Another example of PM_2.5_ aggregate. The top SEM and bottom EDAX images show a particle cluster and the chemical composition (respectively).

**Effect of some of PM_2.5_ to surrounding areas**

Some particles detected with the SEM/AFM revealed some degree of interaction with the Silicon substrate by forming a thin (2 to 10 nm thick) circular layer around them. As mentioned in the manuscript and Figure S14, this layer may be related to small semi-liquid particles attached the bigger one. Despite EDAX analyses were unable to assess the chemical composition of these plateau-like areas, AFM F-Z curves and adhesion/deformation maps, didn't revealed any increase of the adhesion force, suggesting the formation of an inert layer (a liquid would increase the adhesion force (27). Probably oxygen atoms from the PM_2.5_ particle may interact with the underlying Silicon substrate leading to the formation of a thin SiO_2_ layer The formation of thin SiO_2_ layers from oxygen diffusion has been previously reported [30].


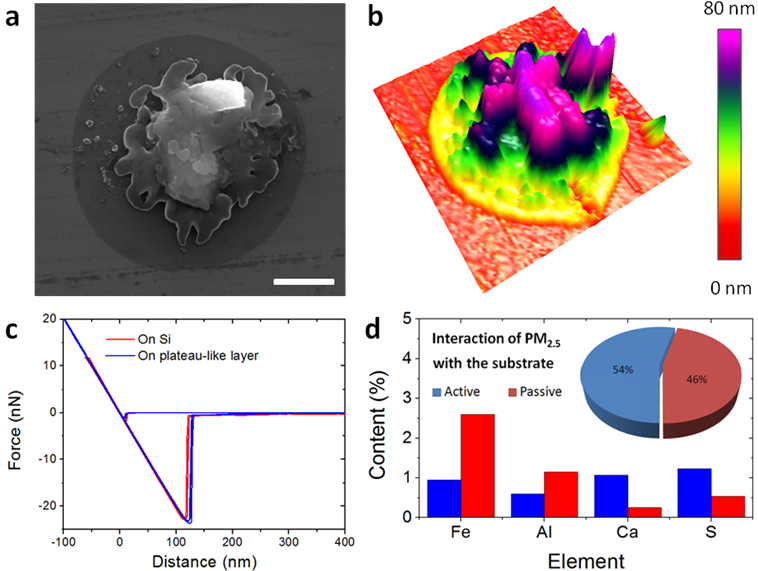


Figure S28: a, SEM image of one PM_2.5_ particle on clean Silicon showing a characteristic dark circular trace around it. **b**, Three dimensional topographic AFM map of a similar particle; the black trace showed heights between 2 and 10 nm. c, Force distance curves on both the Silicon and plateau-like layer. d, EDAX Chemical composition histogramof the particles with trace (active) and without trace (passive); no clear chemical composition difference between the two groups has been observed. The scale bars are 4 μm for (a) and 750 nm for (b).

The EDAX analysis reveal that passive particles are richer in stable metallic oxides like Iron and Aluminum, while passive contain more Cadmium and Sulfur. Probably the particles with stable oxides don't let the oxygen atoms interact with the Silicon substrate, limiting the oxidative effect. Since cell oxidation has been proved to be an important source of toxic effects for cellular tissue, these particles may be specially hazardous.

**Supplementary Information References**

[RS1] User manual of the Quanta 200FEG Scanning Electron Microscope coupled with Energy Dispersive X-ray Spectrometer (SEM-EDAX).

[RS2] Yue, W. S., Li, X. L., Liu, J. F., Li, Y., Yu, X. H., Deng, B., Wan, T. M., Zhang, G. L., Huang, Y. Y., He, W., Hua, W., Shao, L. Y., Li, W. J. & Yang, S. S. Characterization of PM2.5 in the ambient air of Shanghai city by analyzing individual particles. *Science of the Total Environment***368**, 916–925(2006).

[RS3] Gladis, L.-D., Antonio, A.-P.,Arturo, C.-R., Telma, C.-R.,Omar, A.-M.&Rafael, V.-P.Chemical and morphological characterization of PM2.5 collected during MILAGRO campaign using scanning electron microscopy. *Atmospheric Pollution Research***3**, 289-300 (2012).

[RS4] Feng, X. D., Dang, Z., Huang, W. L., Shao, L. Y. &Li, W. J. Microscopic morphology and size distribution of particles in PM2.5 of Guangzhou City. *Journal of Atmospheric Chemistry***64**, 37–51 (2010).

[RS5] Ancelet, T., Davy,P. K.,Trompetter, W. J., Markwitz,A. &Weatherburn, D. C. Carbonaceous aerosols in an urban tunnel. *Atmospheric Environment***45**, 4463-4469(2011).

[RS6] Majumdar, D. & William, S.P. M. P. Chalk dustfall during classroom teaching: particle sizedistribution and morphological characteristics. *Environ Monit Assess***148**, 343-351(2009).

[RS7] Li,Z., Zhao, S.,Edwards,R.,Wang,W. &Zhou, P. Characteristics of individual aerosol particles over Ürümqi Glacier No. 1 ineastern Tianshan, central Asia, China.*Atmospheric Research***99,**57-66(2011).

[RS8] Fernandes,U.&Costa, M. Particle emissions from a domestic pellets-fired boiler.*Fuel Processing Technology***103**, 51-56(2012).

[RS9] Sirghi, L.,Kylián, O., Gilliland, D.,Ceccone, G., Ros, F. Cleaning and hydrophilization of atomic forcemicroscopy silicon probes, *J. Phys. Chem. B*, 2006, 110 (51), pp 25975–25981.
